# A Comparative Effectiveness Meta-Analysis of Drugs for the Prophylaxis of Migraine Headache

**DOI:** 10.1371/journal.pone.0130733

**Published:** 2015-07-14

**Authors:** Jeffrey L. Jackson, Elizabeth Cogbill, Rafael Santana-Davila, Christina Eldredge, William Collier, Andrew Gradall, Neha Sehgal, Jessica Kuester

**Affiliations:** 1 General Internal Medicine, Zablocki VA Medical Center, Milwaukee, Wisconsin, United States of America; 2 Department of Medicine, Western Michigan School of Medicine, Kalamazoo, Michigan, United States of America; 3 Division of Hematology and Oncology, University of Washington, Seattle, Washington, United States of America; 4 Department of Family and Community Medicine, Medical College of Wisconsin, Milwaukee, Wisconsin, United States of America; 5 Department of Pharmacology, Medical College of Wisconsin, Milwaukee, Wisconsin, United States of America; 6 School of Health Sciences, Gollis University, Hergaisa, Somaliland; 7 Medical College of Wisconsin, Milwaukee, Wisconsin, United States of America; Hospital General Dr. Manuel Gea González, MEXICO

## Abstract

**Objective:**

To compare the effectiveness and side effects of migraine prophylactic medications.

**Design:**

We performed a network meta-analysis. Data were extracted independently in duplicate and quality was assessed using both the JADAD and Cochrane Risk of Bias instruments. Data were pooled and network meta-analysis performed using random effects models.

**Data Sources:**

PUBMED, EMBASE, Cochrane Trial Registry, bibliography of retrieved articles through 18 May 2014.

**Eligibility Criteria for Selecting Studies:**

We included randomized controlled trials of adults with migraine headaches of at least 4 weeks in duration.

**Results:**

Placebo controlled trials included alpha blockers (n = 9), angiotensin converting enzyme inhibitors (n = 3), angiotensin receptor blockers (n = 3), anticonvulsants (n = 32), beta-blockers (n = 39), calcium channel blockers (n = 12), flunarizine (n = 7), serotonin reuptake inhibitors (n = 6), serotonin norepinephrine reuptake inhibitors (n = 1) serotonin agonists (n = 9) and tricyclic antidepressants (n = 11). In addition there were 53 trials comparing different drugs. Drugs with at least 3 trials that were more effective than placebo for episodic migraines included amitriptyline (SMD: -1.2, 95% CI: -1.7 to -0.82), -flunarizine (-1.1 headaches/month (ha/month), 95% CI: -1.6 to -0.67), fluoxetine (SMD: -0.57, 95% CI: -0.97 to -0.17), metoprolol (-0.94 ha/month, 95% CI: -1.4 to -0.46), pizotifen (-0.43 ha/month, 95% CI: -0.6 to -0.21), propranolol (-1.3 ha/month, 95% CI: -2.0 to -0.62), topiramate (-1.1 ha/month, 95% CI: -1.9 to -0.73) and valproate (-1.5 ha/month, 95% CI: -2.1 to -0.8). Several effective drugs with less than 3 trials included: 3 ace inhibitors (enalapril, lisinopril, captopril), two angiotensin receptor blockers (candesartan, telmisartan), two anticonvulsants (lamotrigine, levetiracetam), and several beta-blockers (atenolol, bisoprolol, timolol). Network meta-analysis found amitriptyline to be better than several other medications including candesartan, fluoxetine, propranolol, topiramate and valproate and no different than atenolol, flunarizine, clomipramine or metoprolol.

**Conclusion:**

Several drugs good evidence supporting efficacy. There is weak evidence supporting amitriptyline’s superiority over some drugs. Selection of prophylactic medication should be tailored according to patient preferences, characteristics and side effect profiles.

## Introduction

Migraine headaches are common, with a worldwide prevalence ranging between 8 and 18% [1–7]. Migraines cause significant disability [8–11], even during periods between attacks [12], and are responsible for $1 billion in medical costs and $16 billion in lost productivity per year [13,14] in the US alone. The diagnostic criteria for migraine headaches have evolved over time. Currently, the International Headache Society (IHS) diagnostic criteria for migraine includes having at least 5 attacks that last 4–72 hours, that are unilateral, pulsating, moderate or severe in intensity and aggravated by or cause avoidance of routine physical activity and are also accompanied by nausea and/or vomiting, photophobia or phonophobia [15]. IHS further classifies migraine as with or without an aura and as episodic or chronic. Chronic migraine is defined as more than 15 migraine headaches per month for more than 3 months. Chronic migraines result in significantly greater disability than episodic migraines[16].

Treatment of headaches can be either abortive or prophylactic. Abortive treatment provides symptom relief for the acute headache [17,18], while prophylactic treatment aims to reduce the frequency or severity of headaches over time. We focus on prophylactic migraine headache treatment in this manuscript. There are a large number of prophylactic treatment options available; common ones include alpha antagonists, anti-convulsants [19], beta-blockers [20], botulinum-A [21], calcium channel blockers [22], serotonin agonists[23], serotonin reuptake inhibitors (SSRIs) [24] and tricyclic antidepressants (TCAs) [25]. Two emerging prophylactic candidates are angiotensin converting enzymes (ACE) and angiotensin receptor antagonists (ARB). Unfortunately nearly half of males and a third of females who are candidates for prophylactic therapy do not receive it [26]. Selection of prophylactic treatment is tailored on individual patient characteristics, costs and side effects of the available options. However, for patients and their providers, the decision about which prophylactic regimen to use is hampered by the lack of head to head trials comparing the different classes of medications. In addition, previous systematic reviews have focused on single classes of drugs. Two recent systematic reviews that looked more broadly at different drug options have been published. One only included studies since 1999 and did not pool any results, providing qualitative statements about relative treatment effectiveness [27]. Another review analyzed focused only on dichotomous outcomes among patients with episodic migraines and found no difference in likelihood of experiencing at least 50% improvement in headaches between different classes of oral medications [28]. Previous systematic reviews have also had methodological problems. Some combine outcomes from the end of the study, regardless of study duration. This inappropriately combines study results at markedly different time points. This also tends to overstate the strength of the evidence by making it appear that there are more studies contributing data to the results and produces inappropriately narrow confidence intervals. We conducted a meta-analysis asking what is the comparative effectiveness and side effects of the prophylactic treatment of migraine headaches in adults using oral pharmacological medications.

## Materials and Methods

This report closely adheres to the PRISMA guidelines for conducting a systematic review [29]. We searched MEDLINE, EMBASE, the bibliographies of all retrieved articles, published systematic reviews and the Cochrane Database of Clinical Trials for each of the classes of medications (Table 1) through 7 November 2014. The search was conducted independently in duplicate. We included published, randomized clinical trials that evaluated efficacy in reducing the frequency or severity of migraine headaches that were at least 4 weeks in duration among adults. These comparisons could be between active treatment with placebo controls or comparative trials comparing two or more active treatments. We did not include unpublished data as there is no systematic means of searching for it. Because the classification of headache has changed over time [30,31], two authors independently reviewed each included article's headache definition and, where possible, classified it according to the 3rd edition of the International Headache Society (IHS) criteria (ICDH-III) and included only those that could reasonably be defined based on these diagnostic criteria [15]. For headache trials before 2004, we classified trials as focusing on episodic or chronic migraine based on the number of headaches experienced by participants at baseline.

**Table 1:** Search Strategies.

Search Purpose	Search Strategy
Headaches	(headache OR headache disorders OR migrain* OR headache* OR cephalgi* OR cephalalgi* OR tension*)
Randomized controlled trials	(randomized controlled trial [pt] OR controlled clinical trial [pt] OR randomized controlled trials [mh] OR random allocation [mh] OR double-blind method [mh] OR single-blind method [mh] OR clinical trial [pt] OR clinical trials [mh] OR ("clinical trial" [tw]) OR ((singl* [tw] OR doubl* [tw] OR trebl* [tw] OR tripl* [tw]) AND (mask* [tw] OR blind* [tw])) OR (placebos [mh] OR placebo* [tw] OR random* [tw] OR research design [mh:noexp] OR comparative study [mh] OR evaluation studies [mh] OR follow-up studies [mh] OR prospective studies [mh] OR control* [tw] OR prospectiv* [tw] OR volunteer* [tw]) NOT (animals [mh] NOT humans [mh])
Alpha blockers	(“Adrenergic alpha-Antagonists”[MeSH Terms]or clonidine OR tizanidine)
Angiotension converting enzyme inhibitor	“Angiotenin-Converting Enzyme Inhibitors” [mh] OR benzapril OR captopril OR enalapril OR lisinopril OR moexipril OR perindopril OR quinapril OR ramipril OR trandolapril
Angiotension receptor blockers	“Angiotensin Receptor Antagonists” [mh] OR losartan OR irbesartan OR olmesartan OR candesartan OR valsartan OR telmisartan
Anticonvulsants	((anticonvulsants [mh] OR (anticonvulsant* OR antiepileptic* OR acetazolamide OR carbamazepine OR chlormethiazole OR clobazam OR clorazepate OR divalproex OR ethosuximide OR felbamate OR fosphenytoin OR gabapentin OR lamotrigine OR levetiracetam OR mephobarbital OR methsuximide OR midazolam OR oxcarbazepine OR paraldehyde OR pentobarbital OR phenobarbital OR phenytoin OR primidone OR valproate OR tiagabine OR topiramate OR valproic* OR vigabatrin OR zonisamide)
Beta-blocker	adrenergic beta receptor blockaders [mh] OR (alprenolol OR bucindolol OR carteolol OR carvedilol OR labetalol OR nadolol OR penbutolol OR pindolol OR propranolol OR Sotalol OR timolol OR acebutolol OR atenolol OR betaxolol OR bisoprolol OR celiprolol OR esmolol OR metoprolol OR nebivolol)
Calcium channel blocker	(calcium channel blockers/therapeutic use"[mh] OR (amlodipine OR aranidipine OR azelnidipine OR barnidipine OR benidipine OR bepridil OR cilnidipine OR clevidipine OR diltiazem OR efonidipine OR felodipine OR fendiline OR flunarizine OR fluspirilene OR gallopamil OR isradipine OR lacidipine OR lercanidipine OR manidipine OR mibefradil OR nicardipine OR nifedipine OR nilvadipine OR nimodipine OR nisoldipine OR nitrendipine OR pranidipine OR verapamil))
Selective serotonin reuptake inhibitor	serotonin Uptake Inhibitors/therapeutic use [MH] OR (citalopram OR dapoxetine OR escitalopram OR fluoxetine OR fluvoxamine OR indalpine OR paroxetine OR sertraline OR vilazodone OR zimelidine OR venlafaxine OR desvenlafaxine OR duloxetine OR milnacipran OR levomilnacipran OR sibutramine OR bicifadine)
Serotonin agonist (Pizotifen)	Pizotyline [mh] OR pizotifen OR sandomigran
Tricyclic antidepressant	antidepressive agents, tricyclic OR antidepressive$ OR tricyclic$ OR amitriptyline OR amoxapine OR clomipramine OR desipramine OR dibenzepin OR dothiepin OR doxepin OR imipramine OR lofepramine OR nortriptyline OR opipramol OR protriptyline OR trimipramine

* (is the symbol for wild-card in MEDLINE)

Two authors independently abstracted data. Because measures of headache outcomes varied, a priori we followed International Headache Society outcome recommendations by prioritizing abstraction and analysis in this order: 1) headache frequency, 2) a headache index that included frequency, 3) severity or 4) duration [32]. Headache frequency was standardized to number of headaches per month. Whenever possible, we pooled frequency as the number of headaches/month. When not possible, we pooled standardized mean differences between studies, a measure also known as an effect size. By convention, effect sizes greater than 0.8 are considered to be large effect sizes, 0.5–0.8 moderate and 0.2–0.5 small [33]. When missing, variances were calculated from reported mean, sample size and p values [34]; for one non-placebo comparison trial [35] variance was imputed based on sample size and the reported effect size (r^2^ = 0.76) When not explicitly reported, to verify we were using the proper variance, we tested the abstracted data for each article to ensure that the p value reported in the article matched our analysis. This helped insure that standard errors weren’t abstracted as standard deviations, a common error in systematic reviews [36]. In addition, because of reports on the potential for misleading data [37,38], we only accepted data that was unadjusted and that was either based on a true intention to treat analysis or based on the subjects remaining in the trial. We rejected any “modified intention to treat” analyses or analyses subject to other adjustments. We assessed article quality independently and in duplicate, using both component and scales approaches using the Cochrane Risk of Bias Tool [39] and the Jadad scale [40] with good inter-rater agreement (Cochrane ICC: 0.83; Jadad kappa: 0.85). Disagreements were resolved by consensus.

For studies with more than one arm or using a cross-over design, we followed the recommendations of the Cochrane collaboration by pooling the arms into a single arm (if the study reported no differences between arms) or by reducing the sample sizes for cross-over trials by 50% [41]. We abstracted data from each trial at the following time points: baseline, 4, 8, 12, 24, 30 and 36 weeks using the DerSimonian and Laird random effects model [42]. Because of controversy about the accuracy of reporting of off-label use of one of gabapentin [37,38], we relied on data in McCrory’s reanalysis of misleading data presented in one of the studies [43] based on drug company trial data.

The main focus of our analysis is between active treatment and placebo controls. We also included data from comparative effectiveness trials. In addition to direct comparisons between drugs, we also conducted a network meta-analysis [44–47]. In brief, network meta-analysis asks if one drug has a pooled efficacy compared to placebo of X and another drug has a pooled efficacy compared to placebo of Y, are X and Y statistically different? We only included drugs with at least 2 clinical trials and at least 8 weeks in duration, adjusting for duration and for correlation between outcomes reported from the same trial. Because these studies did not always report their outcomes in frequency of headaches, the network meta-analysis was done using standardized mean differences (SMD) rather than weighted mean differences.

Heterogeneity was assessed visually using Galbraith plots [48], and I-square [49].We assessed for small study effects (publication bias) using the methods of Peters [50] for dichotomous outcomes and Eggers [51] for continuous ones. We explored the potential source of heterogeneity using stratified analysis and random-effects meta-regression [52]. These analyses included assessment of the impact of quality, study duration, percentage women, losses to follow-up, and drug dose. All analyses were done using STATA (v 13.1, College Station TX). There was no external funding for this study.

## Results

**Fig 1:**
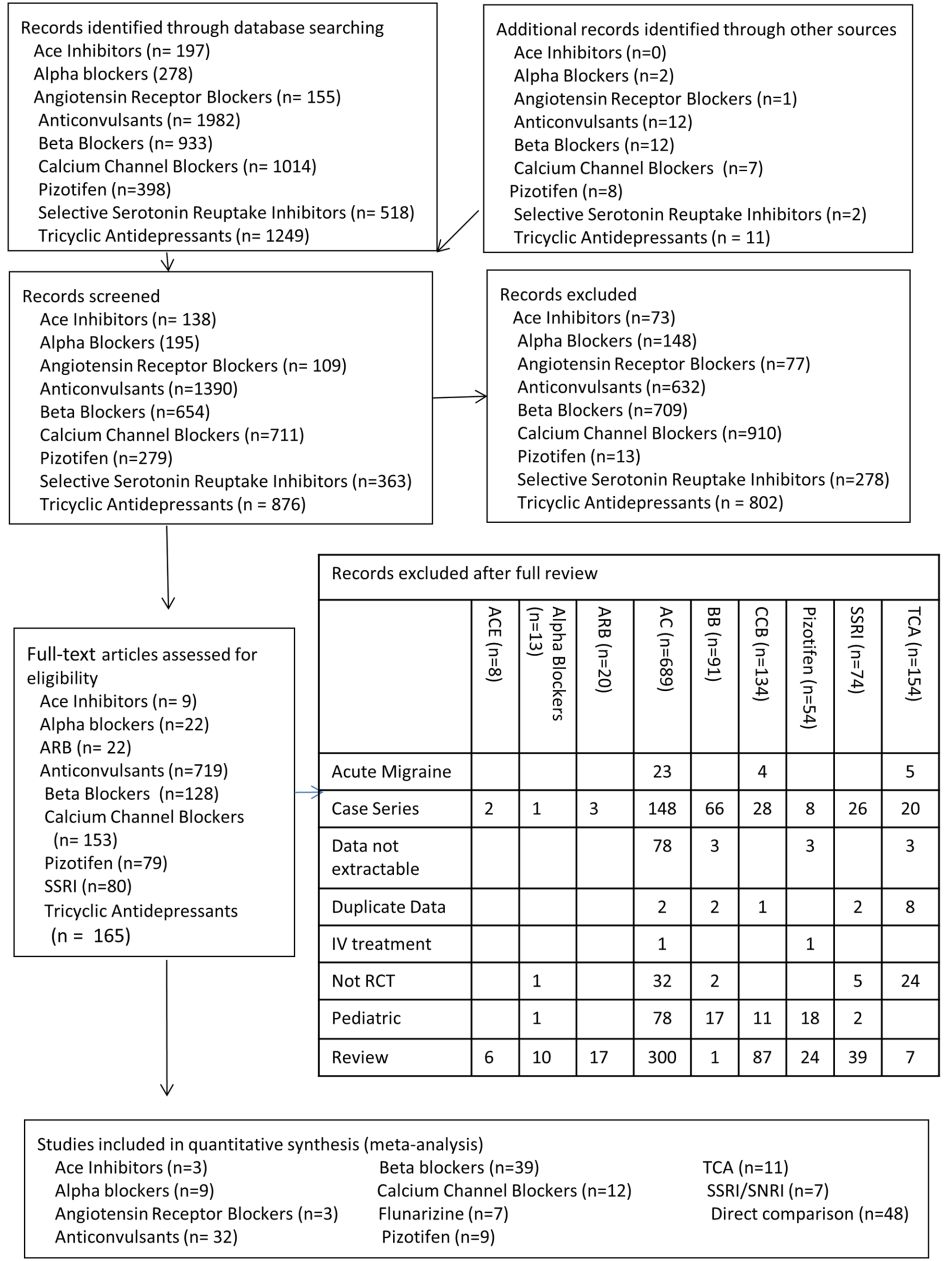
PRISMA Flowchart of study selection.

Individual searches yielded 4789 unique articles: 138 ACE, 195 alpha blockers, 109 ARB, 1391 anticonvulsants, 654 beta blockers, 711 calcium channel blockers, 279 serotonin agonists, 363 SSRI and 876 TCA publications. Application of inclusion criteria (Fig 1) resulted in selecting 179 randomized clinical trials. These included the following placebo controlled trials: 9 alpha blockers [53–61], 3 ACE trials [62–64] 3 ARB [65–67], 33 anticonvulsants [43,68–99], 39 beta-blockers [66,73,100–136], 12 calcium channel blocker [106,137–147], 7 flunarizine [148–154], 6 SSRI [155–160], 1 SNRI [161], 9 serotonin agonists [162–170] and 9 TCA [118,136,171–177] trials. Fifteen of these placebo-controlled trials included more than one active treatment [66,74,106,116,118,131,136,141,163,167,169,170,175,178,179]. In addition, we also include 53 non-placebo controlled comparative effectiveness trials [178–230].

### Placebo Comparisons

Table 2 provides study characteristics of trials investigating prophylactic treatment of episodic migraines (< 15 headaches/month), Table 3 provides details about studies of chronic migraine (>15 headaches/month) and chronic daily headache. There were a total of 15,493 participants in the placebo controlled trials. Studies averaged 112 participants, ranging from 9 to 783. The average patient was 39.2 years old and 78% of subjects were women. Included studies averaged 12 weeks in duration (range 4–82) and had a mean dropout rate of 24%. Thirty nine trials used the 1962 Ad Hoc Committee criteria, seven used the 1969 World Federation of Neurology criteria, forty seven studies used the 1988 International Headache Society criteria, and sixteen the 2004 IHS criteria. Among included trials, most (n = 120) studied episodic migraine headaches with subjects averaging 5.6 headaches per month (range 1.2–11.7). Ten studies focused on subjects with chronic migraine with an average of 18.6 (range 12–24) headaches a month. Six studied chronic daily headaches; the majority of participants (73%) had chronic migraine. Ninety trials (57%) used a parallel-group design, while sixty-six used a crossover design. There were 23 countries contributing studies. Fifty-one trials (46%) were sponsored by industry. Most studies (82%) used frequency as their outcome measure, nineteen (13.7%) used a headache index, two used headache duration and three headache intensity.

**Table 2:** Study characteristics of included randomized trials of treatment of episodic (<15 headaches/month) migraine headaches.

Author, year, Country	Migraine Type	Baseline Headache Frequency	Drugs (mg)	Headache Measure	Study design (washout)	Duration, weeks	Sample size	Dropouts	Age	Female
**ALPHA BLOCKER**										
Adam [53], 1978, UK	Episodic	ns	Clonidine (0.15)	Frequency	Crossover (0)	24	96	27%	37.5	84%
Boison [54], 1978, Denmark	Episodic	ns	Clonidine (0.1)	Frequency	Crossover (0)	8	71	31%	ns	ns
Bredfeldt[55], 1989, USA	Episodic	ns	Clonidine (0.2)	Frequency	Crossover	6	43	30%	ns	80%
Lynggaard [56], 1975, Denmark	Episodic	ns	Clonidine (0.1)	Frequency	Crossover (1)	12	37	26%	34.1	95%
Mondrup[57], 1977, Denmark	Episodic	ns	Clonidine (0.1)	Frequency	Crossover (4)	12	32	34%	35	76%
Ryan [58], 1975, USA	Episodic	ns	Clonidine (0.15)	Frequency	Crossover (2 days)	8	133	ns	41	78%
Shafar [60], 1972, UK	Episodic	8.4	Clonidine (0.1)	Frequency	Crossover	8	65	23%	47.4	84%
Stensrud, 1976, Norway	Episodic	5.8	Clonidine (0.15)	Frequency	Crossover (0)	7	29	7%	43.3	83%
**ANGIOTENSIN ENZYME CONVERTING INHIBITORS**										
Paterna [62] 1992, Italy	Episodic		Captopril (75)	Headache Index	Crossover	16	20	23%	37	81%
Schrader [63] 2001,Norway	Episodic	2.3	Lisinopril (20)	Frequency	Parallel	12	30	5%	41	81%
Sonbolestan [64], 2013, Iran	Episodic	11.3	Enalapril (10)	Frequency	Parallel	8	34	0%	34.4	825
**ANGIOTENSIN RECEPTOR BLOCKER**										
Diener[65] 2009, Germany	Episodic	6.9	Telmisartan (80)	Frequency	Parallel	12	95	5%	47	85%
Stovner[66], 2013, Norway	Episodic	4.8	Candesartan (16), Propranolol (160)	Frequency	Crossover	12	61	15%	37	82%
Tronvik [67](2003) Norway	Episodic	5.7	Candesartan (16)	Frequency	Parallel	12	57	5%	43.2	79%
**ANTICONVULSANTS**										
Brandes [69], 2004, Canada/USA	Episodic	5.7	Topiramate, (50,100,200)	Frequency	Parallel	26	483	46%	38.9	87%
Cady [70], 2009, USA	Episodic	4.4	Carisbamate (100,300,600)	Frequency	Parallel	14	318	30%	41.3	85%
de Tommaso [71], 2007, Italy	Episodic	10.9	Topiramate (100), Levitracetam (1000)	Frequency	Parallel	8	45	16%	37.8	78%
Di’ Trapani [72], 2000, Italy	Episodic	5.2	Gabapentin (1200)	Frequency	Parallel	12	63	0%	ns	52%
Diener [73], 2004 Europe	Episodic	5.1	Topiramate (100, 200) Propranolol (160)	Frequency	Parallel	20	568	37%	40.8	80%
Edwards [75], 2003, USA	Episodic	4.5	Topiramate (200)	Frequency	Parallel	4	70	0%	41.4	97%
Freitag [76], 2002,USA	Episodic	4.2	Divalproex (1000)	Frequency	Parallel	12	237	15%	40.5	79%
Ghose[77], 2002, New Zealand	Episodic (74%) Chronic (26%)	7.6	Vigabatrin (2000)	Frequency	Crossover (4)	12	23	17%	43.6	74%
Gupta [78], 2007, India	Episodic	7.0	Topiramate (50), Lamotrigene (50)	Frequency	Crossover ()	4	60	7%	30	78%
Hering [79],1992. Israel	Episodic	7.7	Valproate (800)	Frequency	Crossover (0)	8	32	9%	34	79%
Jensen [80],1994, Denmark	Episodic	6.6	Valproate (1500)	Frequency	Crossover (4)	12	43	21%	46	86%
Klapper [81],1997, USA	Episodic	5.0	Divalproex (500,100,1500)	Frequency	Parallel	12	176	22%	40.8	89%
Lipton [82], 2011, USA	Episodic	11.7	Topiramate (100)	Frequency	Parallel	26	385	14%	40.3	89%
Mathew [83], 1995, USA	Episodic	6.2	Valproate (750)	Frequency	Parallel	12	107	16%	45.6	78%
Mathew [43], 2001,USA	Episodic	4.9	Gabapentin (2400)	Frequency	Parallel	12	143	39%	40	83%
Rompel [85], 1970, S Africa	Episodic	3.0	Carbamazepin (ns)	Frequency	Crossover ()	6	48	2%	60	69%
Silberstein [87],2004, USA	Episodic	5.5	Topiramate (50,100,200)	Frequency	Parallel	24	487	46%	40.4	89%
Silberstein [88], 2006, USA	Episodic	4.9	Topiramate (200)	Frequency	Parallel	20	211	27%	40.8	86%
Silberstein [90], 2008, USA	Episodic	3–9	Oxcarbazepine(1200)	Frequency	Parallel	15	170	26%	40.5	85%
Silberstein [91], 2013, USA	Episodic	9.2	Gabapentin (1200,1800,2400,3000)	Frequency	Parallel	20	263	29%	39.3–40.6	83%
Steiner [94],1997, UK	Episodic	4.1	Lamotrigine (200)	Frequency	Parallel	12	77	31%	37.2	82%
Stensrud [95],1979, Norway	Episodic	6.3	Clonazepam (1)	Frequency	Crossover ()	4	38	11%	ns	71%
Storey [96],2001, USA	Episodic	4.7	Topiramate (200)	Frequency	Parallel	16	40	13%	38.3	98%
Vahedi [97],2002, France	Episodic	5.0	Acetazolamide (500)	Frequency	Parallel	12	53	34%	39.2	75%
Verma[98], 2013, India	Episodic	5.7	Levetiracetam	Frequency	Parallel	12	65	20%	31.1	73%
**Beta Blockers**										
Ahuja[100], 1985, India	Episodic	7.2	Propranolol (120)	Frequency	Crossover	8	26	ns	ns	46%
Al-Qassab [101], 1993, UK	Episodic	4	Propranolol (80, 160)	Frequency	Crossover	8	45	33%	36	80%
Andersson [102], 1983, Denmark	Episodic	4.9	Metoprolol (200)	Frequency	Parallel	8	71	13%	39.6	85%
Borgesen [103], 1974, Denmark	Episodic	1.8	Propranolol (120)	Frequency	Crossover	12	45	33%	37.6	83%
Briggs [104], 1979, UK	Episodic	6.9	Tomolol (20)	Frequency	Crossover	6	24	4%	ns	71%
Dahlof [105], 1987, Sweden	Episodic	4.3	Propranolol (120)	Frequency	Crossover	4	29	0%	ns	83%
Diener [106], 1996, Germany	Episodic	4	Propranolol (120) Clyclandelate (1200)	Duration	Parallel	20	214	19%	39	78%
Diener [73], 2004, Germany	Episodic	5.1	Propranolol (160)	Frequency	Parallel	20	568	37%	40.8	80%
Ekbom [107], 1972, Sweden	Episodic	11.7	Pindolol (7.5, 15)	Frequency	Parallel	4	30	13%	33.7	87%
Ekbom [108], 1975, Sweden	Episodic	2.2	Alprenolol (400)	Frequency	Parallel	6	33	15%	41.3	82%
Ekbom [109],1977, Sweden	Episodic	>3	Oxprenolol	Headache Index	Crossover (1)	12	34	46%	41.8	89%
Forssman [110], 1976, Sweden	Episodic	6.9	Propranolol (240)	Frequency	Crossover	12	40	20%	37.4	97%
Forssman [111], 1983, Sweden	Episodic	>3	Atenolol (100)	Frequency	Crossover	12	24	17%	40	80%
Freitag [112], 1984, USA	Unclear	ns	Nadolol (160, 240)	Frequency	Parallel	ns	32	ns	ns	81%
Holroyd [113], 2010, USA	Episodic	5.4	Propranolol (180)	Frequency	Parallel	64	232 (35%)	35%	38.2	78%
Johannsson [114], 1987, Sweden	Episodic	>2	Atenolol (100)	Frequency	Crossover	12	Ns	14%	43	70%
Johnson [115], 1986, New Zealand	Episodic	5	Propranolol (240)	Frequency	Crossover	12	29	41%	42	69%
Kangasniemi [116], 1987, Norway	Episodic	4.3	Metoprolol (200)	Frequency	Crossover (4)	8	74	1%	37.5	79%
Langohr [175],1985, Germany	Episodic		Propranolol () Clomipramine (	Frequency	Crossover (4)	12	36	43%	44	74%
Malvea [117], 1973, USA	Episodic	>4	Propranolol (?)	Headache Index	Crossover	6	31	6%	ns	87%
Mathew [118], 1981, USA	Unclear	ns	Propranolol (75)Amitriptyline (75)	Headache Index	Parallel	24	554	22%	38	95%
Mikkelsen [119], 1986, Denmark	Episodic	>3	Propranolol (120)	Frequency	Crossover (0)	12	39	21%	ns	84%
Nadelmann [120], 1986, USA	Unclear	ns	Propranolol (240)	Headache Index	Crossover (0)	6	64	36%	ns	86%
Nanda [121], 1977, Scotland	Episodic	4.8	Acebutolol (800)	Frequency	Crossover (4)	12	43	24%	ns	74%
Pita [123], 1977, Spain	Episodic	5.5	Propranolol (160)	Headache Index	Crossover (0)	8	9	11%	32	78%
Pradalier [124], 1989, France	Episodic	6.1	Propranolol (160)	Frequency	Parallel	12	74	25%	37.5	76%
Sargent [125], 1985, USA	Episodic	>2	Propranolol (120)	Frequency	Parallel	16	161	13%	30	79%
Sjaastad [126], 1972, Norway	Episodic	7.5	Pindolol (7.5)	Frequency	Crossover (3)	4	24	17%	35.3	75%
Standnes [127], 1982, Norway,	Episodic	6.7	Propranolol (160) Timolol (20)	Frequency	Crossover	0	25	28%	ns	80%
Steiner [128], 1988, UK	Episodic	4	Metoprolol (100)	Frequency	Parallel	8	59	19%	37.6	76%
Stellar [129], 1984, USA	Episodic	6.8	Timolol (30)	Frequency	Crossover	6	107	8%	43	72%
Stensrud [130], 1976, Norway	Episodic	6.1	Propranolol (160)Inderal (160)	Headache Index	Crossover (1)	4	20	5%	ns	70%
Stensrud [131], 1980, Norway	Episodic (n = 21)	<15>15	Atenolol (100)Propranolol (160)	Frequency	Crossover (1)	6	21	20%	ns	69%
Tfelt-Hansen [132], 1984, Scandinavia	Episodic	6.0	Timolol (20)Propranolol(160)	Frequency	Crossover (2)	10	96	10%	39.5	74.5%
Van de Ven [133], 1997, Denmark	Episodic	5.5	Bisoprolol (10)	Frequency	Parallel	8	226	14%	38.7	82%
Weber [134], 1972, USA	Unclear	ns	Propranolol (80)	Headache Index	Crossover (0)	12	25	24%	40.5	52%
Wideroe [135], 1974, Norway	Episodic	3	Propranolol (160)	Headache Index	Crossover (0)	12	30	13%	38	87%
Zeigler [136], 1987, USA	Episodic	2–12	Propranolol (240)	Headache Index	Crossover (4)	4	30	ns	38	73%
**Calcium Channel Blockers**										
Nimodipine European Migraine (with aura) Trial [137], 1989, EU	Episodic	3.3	Nimodipine (120)	Frequency	Parallel	12	89	19%	33.8	79%
Nimodipine European Migraine (Without aura) trial (1989) [138], EU	Episodic	4.4	Nimodipine (120)	Frequency	Parallel	12	192	16%	38.1	78%
Ansell [139], 1988, UK	Episodic	>2	Nimodipine (120)	Headache Index	Parallel	12	68	16%	ns	71%
Gelmers [140], 1983, Netherlands	Episodic	9.1	Nimodipine (120)	Headache Index	Parallel	12	60	17%	30	62%
Havanka-Kanniainen [141], 1985, Finland	Episodic	7.9	Nimodipine (120)	Frequency	Crossover (0)	8	33	12%	33	85%
Leandri [142], 1990, Italy	Episodic	4.3	Nicardipine (40)	Frequency	Crossover (ns)	8	35	15%	ns	ns
Markley [143], 1984, USA	Episodic	3.4	Verapamil (240)	Frequency	Crossover (ns)	8	20	30%	33	86%
McArthur [144], 1989, USA	Episodic	2.3	Nifedipine (90)	Frequency	Crossover (1)	12	24	42%	ns	ns
Shukla [145], 1995, UK	Episodic	10.4	Nifedipine (15)	Frequency	Crossover (ns)	6	36	22%	22.8	50%
Solomon [146], 1983, USA	Episodic	6.7	Verapamil (320)	Frequency	Crossover (ns)	6	12	52%	38	78%
Stewart [147], 1988, Canada	Episodic	6.3	Nimodipine (120)	Frequency	Parallel	8	37	19%	ns	ns
**Flunarizine**										
Diamond [148], 1992, USA	Episodic	4.3	Flunarizine (10)	Frequency	Parallel	20	143	8%	34.8	74%
Frenken [149], 1984, Netherlands	Episodic	3.6	Flunarizine (10)	Frequency	Parallel	12	35	0%	NS	83%
Louis [150], 1981, Belgium	Episodic	1.2	Flunarizine (10)	Frequency	Parallel	12	58	0%	29	50%
Mendenopoulos [151], 1985, Greece	Episodic	4	Flunarizine (10)	Headache Index	Parallel	12	20	0%	44	80%
Pini [152], 1986, Italy	Episodic	9.9	Flunarizine (20)	Headache Index	Parallel	4	18	0%	40.2	83%
Sorensen [153], 1986, Denmark	Episodic	3	Flunarizine (10)	Frequency	Crossover (4)	16	29	7%	40	79%
Thomas [154], 1991, India	Episodic	6.7	Flunarizine (10)	Headache Index	Crossover (2)	12	29	48%	30.5	87%
**Selective Serotonin Reuptake Inhibitor**										
Adly [155], 1993, USA.	Episodic	>4	Fluoxetine (40)	Headache Index	Parallel	10	32	44%	37.5	83%
d'Amato [156], 1999, Italy.	Episodic	1–4	Fluoxetine (20)	Headache Index	Parallel	20	52	0%	37.6	63%
Landy [157], 1998, USA.	Episodic	>2	Sertraline (50)	Headache Index	Parallel	8	27	41%	36	93%
Steiner [159], 1998, UK.	Episodic	3.9	s-Fluoxetine (40)	Frequency	Parallel	12	53	32%	37	75%
Zeeberg [160], 1981, Sweden	Episodic	3.5	Femoxitine (300)	Headache Index	Parallel	12	59	ns	ns	ns
**Serotonin Norepinephrine Reuptake Inhibitor**										
Ozyalcin [161], 2004, Turkey	Episodic	2.3	Venlafaxine (75, 150)	Frequency	Parallel	8	60	17%	36.5	83%
**Serotonin Agonist**										
Arthur [162], 1971, New Zealand	Episodic	8.1	Pizotifen (3.0)	Frequency	Crossover (ns)	4	63	17%	ns	ns
Bellavance [163], 1990, Canada	Episodic	6.7	Pizotifen (1.5)	Frequency	Parallel	12	176	14%	32.5	79%
Carroll [164], 1975, UK	Episodic	>3	Pizotifen (3.0)	Headache Index	Crossover (2)	4	27	48%	ns	ns
Cleland [165], 1997, UK	Episodic	3.4	Pizotifen (2.0)	Frequency	Crossover (ns)	12	130	32%	40.5	63%
Hughes [166], 1971, UK	Episodic	9.1	Pizotifen (0.5)	Frequency	Crossover (ns)	12	26	0%	ns	81%
Kangasniemi [167], 1979, Finland	Episodic	4.3	Pizotifen (1.5)	Frequency	Crossover (0)	7	50	22%	36	80%
Lance, 1968			Pizotifen							
Lawrence [168], 1977, UK	Episodic	>4	Pizotifen (1.5)	Headache Index	Parallel	12	36	14%	ns	79%
Osterman [169], 1977, Sweden	Episodic	5.1	Pizotifen (0.5)	Frequency	Crossover (2)	8	30	10%	37	70%
Ryan [170], 1968, USA	Episodic	8.9	Pizotifen (4)	Frequency	Crossover (ns)	4	62	ns	ns	ns
**Tricyclic Antidepressants**										
Couch [241], 1976, USA	Episodic	6.9	Amitriptyline (100)	Headache Index	Parallel	4	73	36%	NS	64%
Couch [171], 1979	Episodic	6.9	Amitriptyline (100)	Frequency	Parallel	8	162	38%	NS	85%
Couch [172], 2011, USA	Episodic	7.6	Amitriptyline (100)	Frequency	Parallel	16	391	51%	34.9	81%
Gomersall [173], 1973, UK	Episodic	2.7	Amitriptyline (60)	Frequency	Crossover (0)	26	20	20%	42	75%
Jacobs [174], 1972, UK	Episodic	3.3	Opipramol (75)	Frequency	Parallel	12	27	43%	42	78%
Langohr [175], 1985, Germany	Episodic	>4	Clomipramine (100)	Frequency	Crossover (4)	4	36	43%	44	67%
Mathew [118], 1981, USA	Unclear	Unclear	Amitriptyline (75)	Headache Index	Parallel	24	554	22%	38	95%
Noone [177], 1980, UK	Episodic	6	Clomipramine (30)	Frequency	Crossover	4	10	50%	Ns	70%
Ziegler [136], 1987, USA	Episodic	2–12	Amitriptyline (100)	Headache Index	Crossover (1)	8	30	0%	38	73%

**Table 3:** Study characteristics of included randomized trials of treatment of chronic (≥15 headaches/month) migraine headaches.

**Alpha Blocker**										
Saper [59], 2002, USA	Chronic	ns	Tizanidine (24)	Headache Index	Parallel	12	136	32%	40	79%
**Anticonvulsant**										
Diener [74], 2007, Italy	Chronic	15.9	Topiramate (100)	Frequency	Parallel	24	59	36%	46.1	75%
Mei [84], 2006, Italy	Chronic	24	Topiramate (100)	Frequency	Parallel	12	50	42%	45.8	69%
Silberstein [89], 2007, USA	Chronic	17.0	Topiramate (100)	Frequency	Parallel	16	306	46%	38.2	85%
Silvestrini [92], 2003, Italy	Chronic	20	Topiramate (50)	Frequency	Parallel	8	28	0%	43.5	64%
Yurekli, 2008, Turkey	Chronic	22	Valproate (1000)	Frequency	Parallel	12	29	9%	40.4	83%
**Beta-Blockers**										
Palferman [122], 1983, UK	Chronic	12.1	Propranolol (120)	Frequency	Crossover(?)	8	22	39%	37.8	73%
Stensrud [131], 1980, Norway	Chronic	>15	Atenolol (100) Propranolol (160)	Frequency	Crossover (1)	6	7	20%	ns	69%
**Chronic Daily Headache (>15 Headaches/Month)**										
**Anticonvulsants**										
Beran[68], 2010, Australia	Chronic Daily Headache	19.6	Levetiracetam (3000)	Frequency	Crossover (1)	11	96	30%	48.8	53%
Sarchielli [86], 2014, Italy	Chronic Daily Headache (Medication overuse)	21.8	Valproate (800)	Frequency	Parallel	24	88	17%	ns	90%
Spira [93], 2003,Australia	Chronic Daily Headache	27.4	Gabapentin (2400)	Frequency	Crossover (1)	8	133	17%	43	69%
Yurekli [99], 2008, Turkey	Chronic Daily	22.7	Valproate (1000)	Frequency	Parallel	12	29	0%	40.4	83%
**Selective Serotonin Reuptake Inhibitor**										
Saper [158], 1994, USA	Chronic Daily	>16	Fluoxetine (40)	Frequency	Parallel	12	111	5	36.5	87%
Stensrud [131], 1980, Norway	Episodic (n = 21)Chronic (n = 7)	<15>15	Atenolol (100) Propranolol (160)	Frequency	Crossover (1)	6	35	20%	ns	69%

Overall, the studies varied in quality. Quality ratings for placebo controlled trials are given in Table 4. By Jadad criteria, 34% of studies had scores ≤ 3.0, suggesting low quality, 39% had scores between 3 and 5 consistent with modest quality and only 37% had scores ≥ 5 suggesting high quality. Only 36% used an intention to treat analysis, 27% assessed compliance, 26% had concealed allocation, and 51% had adequate blinding. There was no difference in the overall effect sizes for placebo controlled trials using Jadad criteria as a scale (p = 0.44) or when coded as high, modest or low quality (p = 0.37), or when assessed by most of the specific Jadad or Cochrane Risk of Bias quality characteristics (compliance p = 0.59; blinding p = 0.36; adequacy of blinding p = 0.50, industry sponsorship p = 0.52; incomplete outcome reporting p = 0.96, reporting of withdrawals p = 0.24). However, trials which had inadequate concealed allocation had significantly (p = 0.02) higher reported effects (SMD: -0.52, 95% CI: -0.63 to -0.41) than those who had concealed allocation (SMD: -0.26, 95% CI: -0.34 to 0.17).

**Table 4:** Quality Ratings of included placebo controlled trials.

			Cochrane Risk of Bias						
Study	Jadad Score (0–8)	Intention to Treat	Adequate sequence generation	Adequate concealed allocation	Adequate Blinding	Incomplete outcome data addressed	Free of selective outcome reporting	Free of “other” bias	Industry sponsored
**EPISODIC MIGRAINES**									
**Alpha Blockers**									
Adam, 1978, UK	2	No	Unclear	Unclear	Unclear	Unclear	Unclear	Unclear	No
Boison, 1978, Sweden	2	No	Unclear	Unclear	Unclear	Unclear	Unclear	Unclear	No
Bredfeldt, 1989, USA	5	No	Unclear	Unclear	Yes	Unclear	Unclear	Unclear	Yes
Lynggaard, 1975, Denmark	4	No	Unclear	Unclear	Unclear	Unclear	Unclear	Unclear	Unclear
Mondrup, 1977, Denmark	6	No	Unclear	Unclear	Yes	Unclear	Unclear	Unclear	No
Ryan, 1975, USA	1	No	Unclear	Unclear	Unclear	Unclear	Unclear	Unclear	No
Shafar, 1972, UK	4	No	Unclear	Unclear	Unclear	Unclear	Unclear	Unclear	Yes
Stensrud, 1976, Norway	0	No	Unclear	Unclear	Unclear	Unclear	Unclear	Unclear	No
**Antiogensin Enzyme Converting Inhibitors**									
Schrader (2001), Norway, Lisinopril	6	Yes	Unclear	Unclear	Yes	Unclear	Unclear	Unclear	Yes
**Angiotensin Receptor Blocker**									
Diener (2009), Germany, Telmisartan	3	No	Unclear	Unclear	Unclear	Unclear	Unclear	Unclear	Yes
Trovnik (2003) Norway, Candesartan	8	Yes	Yes	Yes	Yes	Unclear	Unclear	Unclear	Yes
**Anticonvulsants**									
Brandes, 2004, Canada/USA, Topiramate	8	Yes	Yes	Yes	Yes	Yes	Yes	Unclear	Yes
Cady, 2009, USA, Carisbamate	8	Yes	Yes	Yes	Yes	Unclear	Unclear	Unclear	Yes
de Tommaso, 2007, Italy, Topiramate, Levitracetam	4	No	Unclear	Unclear	Unclear	Unclear	Unclear	Unclear	Unclear
Di’ Trapani, 2000, Italy, Gabapentin	4	Yes	Unclear	Unclear	Unclear	Yes	Unclear	Unclear	Unclear
Diener, 2004 Europe, Topiramate, Propropranolol	6	Yes	Unclear	Unclear	Unclear	Yes	Yes	Unclear	Yes
Diener, 2007, Italy, Topiramate	8	No	Yes	Yes	Yes	Yes	Unclear	Unclear	Unclear
Edwards, 2003, USA, Topiramate	4	Yes	Unclear	Unclear	Unclear	Yes	Unclear	Unclear	Yes
Freitag, 2002, USA, divalproex	8	Yes	Yes	Yes	Yes	Yes	Unclear	Unclear	Yes
Gupta, 2007, India, Topiramate	8	Yes	Yes	Yes	Yes	Yes	Unclear	No	Unclear
Hering 1992, Israel, Valproate	4	No	Unclear	Unclear	Unclear	Unclear	Unclear	Unclear	Unclear
Jensen,1994, Denmark, Valproate	6	No	Unclear	Unclear	Yes	Unclear	Unclear	Unclear	Yes
Klapper,1997, USA, Divalproex	4	Yes	Unclear	Unclear	Unclear	Unclear	Unclear	Unclear	Yes
Lipton, 2011, USA, Topiramate	8	Yes	Yes	Yes	Yes	Yes	No	Unclear	Yes
Mathew, 1995, USA, Valproate	6	No	Unclear	Unclear	Yes	Unclear	Unclear	Unclear	Yes
Mathew, 2001, USA, Gabapentin	6	Yes	Yes	Unclear	Unclear	Unclear	Unclear	Unclear	Yes
Rompel, 1970, S Africa, Carbamazepine	5	No	Yes	Yes	Yes	Yes	Yes	Unclear	Yes
Silberstein,2004, USA, Topiramate	6	Yes	Yes	Yes	Unclear	Yes	Unclear	Unclear	Yes
Silberstein, 2006, USA, Topiramate	4	Yes	Unclear	Unclear	Unclear	Unclear	Unclear	Unclear	Yes
Silberstein, 2008, USA, Oxcarbazepine	8	Yes	Yes	Yes	Yes	Yes	Unclear	Unclear	Yes
Steiner,1997, UK, Lamotrigine	6	No	Unclear	Unclear	Yes	Unclear	Unclear	Unclear	Unclear
Stensrud,1979, Norway, Clonazepam	2	No	Unclear	Unclear	Unclear	Unclear	Unclear	Unclear	Unclear
Storey,2001, USA, Topiramate	4	No	Unclear	Unclear	Unclear	Unclear	Unclear	Unclear	Yes
Vahedi,2002, France, Acetazolamide	6	Yes	Yes	Yes	Yes	Yes	Yes	Unclear	Yes
**Beta Blockers**									
Ahuja, 1985, India, Propanolol	2	No	Unclear	Unclear	Unclear	Unclear	Unclear	Unclear	Yes
Al-Qassab, 1993, England, Propanolol	3	No	Unclear	Unclear	Yes	Unclear	Unclear	Unclear	Yes
Andersson, 1983, Denmark, Metoprolol	2	Yes	Unclear	Unclear	Yes	No	Unclear	Unclear	Unclear
Borgesen, 1974, Denmark, Propranolol	4	No	Unclear	Unclear	Yes	No	No	No	Unclear
Dahlof, 1987, Sweden, Propranolol	5	Yes	Unclear	Unclear	Yes	No	No	No	Unclear
Diener, 1996, Germany, Propranolol, Clyclandelate	4	Yes	Unclear	Unclear	Unclear	Unclear	Unclear	Unclear	Unclear
Diener, 2004, Germany, Propranolol	6	Yes	Unclear	Unclear	Unclear	Yes	Yes	Unclear	Yes
Ekbom, 1972, Sweden, Pindolol	2	No	Unclear	Unclear	Unclear	Unclear	Unclear	Unclear	Unclear
Ekbom, 1975, Sweden, Alprenolol	2	No	Unclear	Unclear	Unclear	Yes	Unclear	Unclear	Yes
Forssman, 1976, Sweden, Propranolol	6	No	Unclear	Unclear	Yes	Unclear	Unclear	Unclear	Yes
Forssman, 1983, Sweden, Atenolol	2	No	Unclear	Unclear	Unclear	Unclear	Unclear	Unclear	Yes
Freitag, 1984, USA, Nadolol	3	Yes	Unclear	Unclear	Yes	Unclear	Unclear	Unclear	Unclear
Holroyd, 2010, USA, Propanolol	6	Yes	Yes	Yes	Yes	Yes	Yes	Unclear	Unclear
Johannsson, 1987, Sweden, Atenolol	2	No	Unclear	Unclear	Unclear	Unclear	Unclear	Unclear	Unclear
Johnson, 1986, New Zealand, Propanolol	3	No	Unclear	Unclear	Yes	Unclear	Unclear	Unclear	Unclear
Kangasniemi, 1987, Norway, Metoprolol	0	4	Yes	Unclear	Unclear	Unclear	Yes	Unclear	Unclear
Malvea, 1973, USA, Propanolol	4	Yes	Unclear	Unclear	Unclear	Yes	Unclear	Unclear	Unclear
Mathew, 1981, USA, Propanolol, Amitriptyline	2	No	Unclear	Unclear	No	No	No	Yes	Unclear
Mikkelsen, 1986, Denmark, Propanolol	6	No	Unclear	Unclear	Unclear	Unclear	Unclear	Unclear	Yes
Nadelmann, 1986, USA, Propanolol	6	No	Unclear	Unclear	Yes	Unclear	Unclear	Unclear	Yes
Nanda, 1977, Scotland, Acebutolol	2	No	Unclear	Unclear	Unclear	No	Unclear	Unclear	Yes
Pita, 1977, Spain, Propranolol	6	No	No	Unclear	Yes	Yes	Unclear	Unclear	Yes
Pradalier, 1989, France, Propranolol	5	Yes	Unclear	Unclear	Yes	Unclear	Unclear	Unclear	Unclear
Sargent, 1985, USA, Propranolol	4	No	Unclear	Unclear	Unclear	Unclear	Unclear	Unclear	Unclear
Sjaastad, 1972, Norway, Pindolol	5	No	Unclear	Unclear	Yes	Unclear	Unclear	Unclear	Unclear
Standnes, 1982, Norway, Propranolol, Timolol	4	No	Unclear	Unclear	Yes	Unclear	Unclear	Unclear	Yes
Steiner, 1988, UK, Metoprolol	6	No	Unclear	Unclear	Yes	No	No	Unclear	Unclear
Stellar, 1984, USA, Timolol	7	No	Yes	Unclear	Yes	No	Unclear	Unclear	Unclear
Stensrud, 1976, Norway, Propranolol, Inderal	5	No	Unclear	Unclear	Yes	Unclear	Unclear	Unclear	Yes
Tfelt-Hansen, 1984, Scandinavia, Timolol, Propranolol	6	No	Unclear	Unclear	Yes	Unclear	Unclear	Unclear	Unclear
Van de Ven, 1997, Denmark, Bisoprolol	4	Yes	Unclear	Unclear	Unclear	Unclear	Unclear	Unclear	Unclear
Wideroe, 1974, Norway, Propranolol	4	No	Unclear	Unclear	Yes	No	Unclear	Unclear	Yes
Zeigler, 1987, USA, Propranolol	3	No	Unclear	Unclear	Yes	Yes	Unclear	Unclear	No
**Calcium Channel Blockers**									
Ansell, 1988, UK, Nimodipine	3	No	Unclear	Unclear	Unclear	Unclear	Unclear	Unclear	Unclear
Gelmers, 1983, Netherlands, Nimodipine	4	No	Unclear	Unclear	Unclear	Unclear	Unclear	Unclear	Unclear
Havanka-Kanniainen, 1985, Finland, Nimodipine	4	No	Unclear	Unclear	Unclear	Unclear	Unclear	Unclear	Yes
Leandri, 1990, Italy, Nicardipine	3	No	Unclear	Unclear	Yes	Unclear	Unclear	Unclear	Yes
Markley, 1984, USA, Verapamil	3	No	Unclear	Unclear	Unclear	Unclear	Unclear	Unclear	Unclear
McArthur, 1989, USA, Nifedipine	3	No	Unclear	Unclear	Unclear	Unclear	Unclear	Unclear	Yes
Nimodipine European Migraine (with aura) Trial, 1989, EU, Nimodipine	2	No	Unclear	Unclear	Unclear	Unclear	Unclear	Unclear	Unclear
Nimodipine European Migraine (Without aura) trial (1989), EU, Nimodipine	5	Yes	Unclear	Unclear	Yes	Unclear	Unclear	Unclear	Unclear
Shukla, 1995, UK, Nifedipine	5	No	Unclear	Unclear	Yes	Unclear	Unclear	Unclear	Unclear
Solomon, 1983, USA, Verapamil	5	No	Unclear	Unclear	Yes	No	No	Unclear	Yes
Stewart, 1988, Canada, Nimodipine	2	No	Unclear	Unclear	Unclear	Unclear	Unclear	Unclear	Unclear
**Flunarazine**									
Diamond, 1993, USA,	2	No	Unclear	Unclear	Unclear	Unclear	Unclear	Unclear	Unclear
Frenken, 1984, Netherlands, Flunarizine	6	Yes	Unclear	Unclear	Yes	Yes	Unclear	Unclear	Yes
Louis, 1981, Belgium, Flunarizine	5	Yes	Unclear	Unclear	Yes	Yes	Yes	Unclear	No
Mendenopoulos, 1985, Greece, Flunarizine	7	Yes	Yes	Yes	Yes	Yes	Unclear	Unclear	Yes
Pini, 1986, Italy, Flunarizine	2	No	Unclear	Unclear	Unclear	Unclear	Unclear	Unclear	Unclear
Sorensen, 1986, Denmark, Flunarizine	4	No	Unclear	Unclear	Unclear	Unclear	Unclear	Unclear	Unclear
Thomas, 1991, India, Flunarizine	3	No	Unclear	Unclear	Yes	Unclear	Unclear	Unclear	Unclear
**Selective Serotonin Reuptake Inhibitors**									
Adly, 1993, USA, Fluoxetine	2	No	Unclear	Yes	Unclear	No	No	Unclear	Unclear
d'Amato, 1999, Italy, Fluoxetine	5	No	Unclear	Unclear	Yes	No	No	Unclear	Unclear
Landy, 1998, USA, Sertaline	3	No	Unclear	Unclear	Unclear	No	No	Unclear	No
Steiner, 1998, UK, s-Fluoxetine	6	No	Unclear	Unclear	Yes	No	No	Unclear	Unclear
Zeeberg, 1981, Sweden, Femoxitine	2	No	No	No	No	Unclear	Unclear	Unclear	Unclear
**Serotonin Norepinephrine Reuptake Inhibitor**									
Ozyalcin, 2004, Turkey, Venlafaxine	3	No	Unclear	Unclear	Unclear	No	No	Unclear	Yes
**Serotonin Agonist (Pizotifen)**									
Arthur, 1971, New Zealand, Pizotifen	2	No	Unclear	Unclear	Unclear	Unclear	Unclear	Unclear	Yes
Bellavance, 1990, Canada, Pizotifen	4	No	Unclear	Unclear	Unclear	Unclear	Unclear	Unclear	Unclear
Carroll, 1975, UK, Pizotifen	4	No	Unclear	Unclear	Unclear	Unclear	Unclear	Unclear	Unclear
Cleland, 1997, UK, Pizotifen	4	No	Unclear	Unclear	Unclear	Unclear	Unclear	Unclear	Unclear
Hughes, 1971, UK, Pizotifen	4	Yes	Unclear	Unclear	Unclear	Unclear	Unclear	Unclear	Unclear
Kangasniemi, 1979, Finland, Pizotifen	4	Yes	Unclear	Unclear	Unclear	Yes	Unclear	Unclear	Unclear
Lance, 1968, Pizotifen									
Lawrence, 1977, UK, Pizotifen	4	No	Unclear	Unclear	Yes	Unclear	Unclear	Unclear	Unclear
Osterman, 1977, Sweden, Pizotifen	5	No	Unclear	Unclear	Yes	Unclear	Unclear	Unclear	Unclear
Ryan, 1968, USA, Pizotifen	5	No	Unclear	Unclear	Unclear	Unclear	Unclear	Unclear	Unclear
**Tricyclic Antidepressants**									
Couch, 1976, USA, Amitriptyline	3	No	Unclear	Unclear	Yes	No	Unclear	Yes	Yes
Couch, 1979, Amitriptyline	6	No	Unclear	Unclear	Yes	No	Unclear	Unclear	Yes
Couch, 2011, USA, Amitriptyline	8	No	Yes	Yes	Yes	No	Unclear	Unclear	Yes
Gomersall, 1973, UK, Amitriptyline	3	No	Unclear	Unclear	Unclear	No	No	Yes	Yes
Jacobs, 1972, UK, Opipramol	4	No	Unclear	Yes	Yes	No	No	Yes	Yes
Langohr, 1985, Germany, Clomipramine	4	No	Unclear	Unclear	Unclear	No	No	Yes	Yes
Morland, 1979, Norway, Doxepin	3	No	Unclear	Unclear	Unclear	No	Unclear	Yes	Unclear
Noone, 1980, UK, Clomipramin	4	No	Yes	Yes	Unclear	No	Unclear	Yes	Yes
Ziegler, 1987, USA, Amitriptyline	3	No	Unclear	Unclear	Yes	Yes	Unclear	Unclear	No
**CHRONIC MIGRAINES**									
**Alpha-blockers**									
Saper, 2002, USA	6	No	Unclear	Unclear	Yes	Unclear	Unclear	Unclear	Yes
**Anticonvulsants**									
Diener, 2007, Italy, Topiramate	8	No	Yes	Yes	Yes	Yes	Unclear	Unclear	Unclear
Mei, 2006, Italy, Topiramate	4	Yes	Unclear	Unclear	Unclear	Unclear	Unclear	Unclear	Unclear
Silberstein, 2007, USA, Topiramate	8	Yes	Yes	Yes	Yes	Yes	Unclear	Unclear	Yes
Silvestrini, 2003, Italy, Topiramate	4	Yes	Unclear	Unclear	Unclear	Yes	Unclear	Unclear	Unclear
**Beta Blockers**									
Palferman, 1983, UK, Propranolol	3	No	Unclear	Unclear	Unclear	Unclear	Unclear	Unclear	Unclear
**CHRONIC DAILY HEADACHE**									
**Anticonvulsants**									
Beran, 2010, Australia, Levetiracetam	5	No	Yes	Yes	Unclear	Unclear	Unclear	Unclear	Yes
Spira, 2003, Australia, Gabapentin	4	No	Unclear	Unclear	Unclear	Unclear	Unclear	Unclear	Unclear
Yurekli, 2008, Turkey, Valproate	4	No	Unclear	Unclear	Yes	Yes	Unclear	Unclear	Unclear
**Selective Serotonin Reuptake Inhibitors**									
Saper, 1994, USA, Fluoxetine	8	No	Yes	Yes	Yes	Unclear	Unclear	Unclear	Yes
**MIXED (CHRONIC + EPISODIC)**									
**Anticonvulsants**									
Ghose, 2002, New Zealand, Vigabatrin	4	No	Unclear	Unclear	Unclear	Unclear	Unclear	Unclear	Unclear
**Beta Blockers**									
Stensrud, 1980, Norway, Atenolol	5	No	Unclear	Unclear	Yes	Unclear	Unclear	Unclear	Yes
**Unclear Migraine Headache Type**									
**Beta Blockers**									
Freitag, 1984, USA, Nadolol	3	Yes	Unclear	Unclear	Yes	Unclear	Unclear	Unclear	Unclear
Mathew, 1981, USA, Propranolol, Amitriptyline	2	No	Unclear	Unclear	No	No	No	Yes	Unclear
Nadelmann, 1986, USA, Propranolol	6	No	Unclear	Unclear	Yes	Unclear	Unclear	Unclear	Yes
Weber, 1972, USA, Propranolol	3	No	Unclear	Unclear	Unclear	Unclear	Unclear	Unclear	Yes
**Selective Serotonin Reuptake Inhibitors**									
Saper, 1994, USA, Fluoxetine	8	No	Yes	Yes	Yes	Unclear	Unclear	Unclear	Yes
**Tricyclic Antidepressants**									
Mathew, 1981, USA, Amitriptyline	2	No	Unclear	Unclear	No	No	No	Yes	Unclear

#### Alpha-blockers

**Fig 2:**
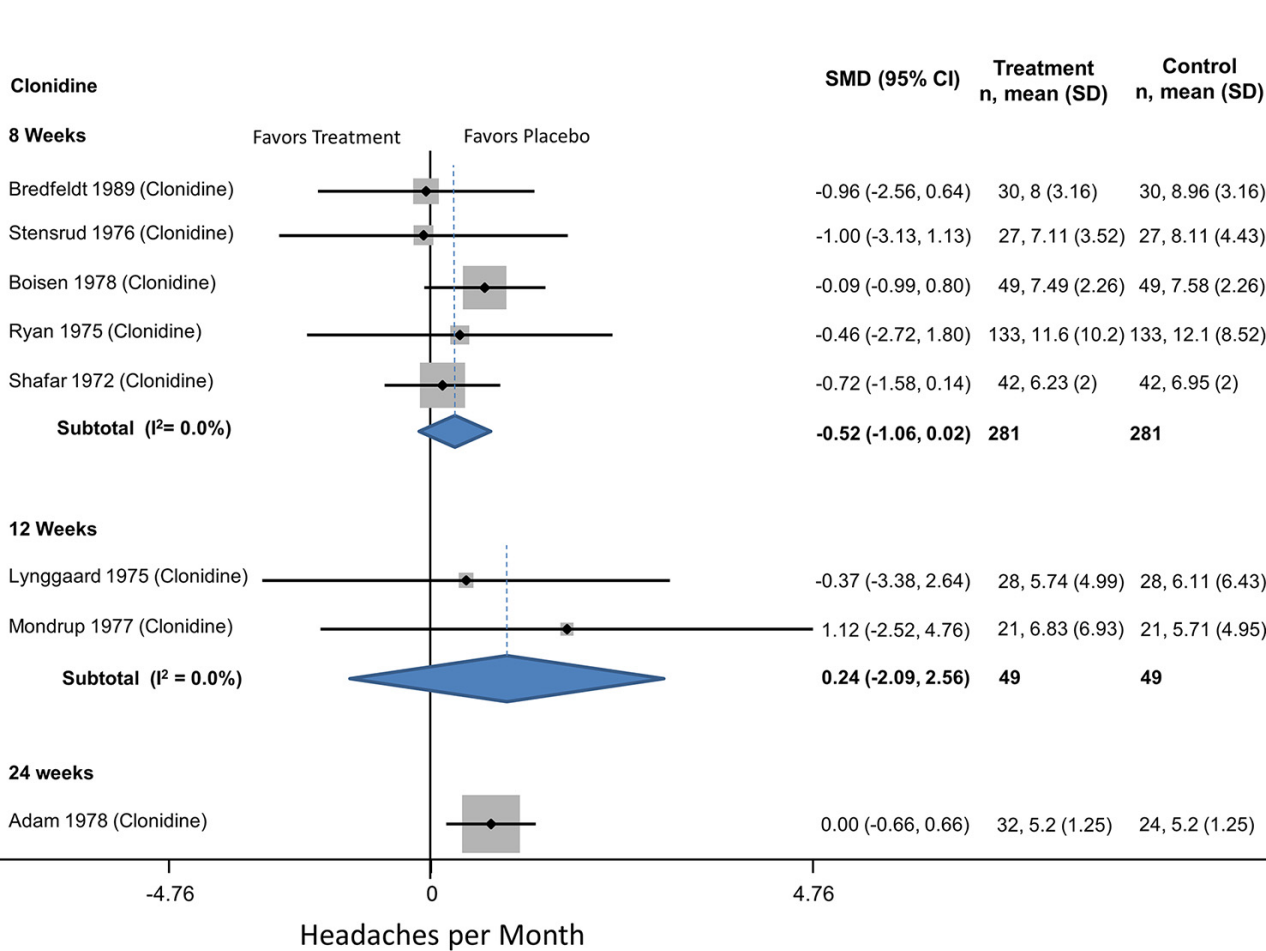
Alpha blockers compared to placebo for episodic migraine headaches.

There were 9 trials comparing alpha blockers to placebo with a total of 4590 participants who averaged 39.3 (range 12–76) years in age with 84% women (Table 2). All of the studies measured headache frequency. Eight of these trials focused on episodic migraine headaches; all studied clonidine. One trial focused on chronic migraines using tizanidine. The average rate of withdrawals was 32%. Studies averaged 11 weeks (range 4–82) with a mean of 71.3 participants (range 11–67). At no time point was clonidine more effective than placebo for episodic migraines (Table 5, Fig 2) and tizanidine was no more effective than placebo for chronic migraine headaches (Table 6). None of these trials reported on the likelihood of a 50% reduction in headaches.

**Table 5:** Placebo Controlled Randomized Clinical Trials of Continuous Outcomes among patients with episodic migraines (<15 headaches/month).

Drug	Time Point (weeks)	Metric	Study (Year)	Pooled Mean Difference (95% CI)	Heterogeneity
**Alpha Blockers**					
Clonidine	8	Headaches/month	Boisen (1978)	-0.09 (-0.99 to 0.80)	
	8	Headaches/month	Bredfeldt (1989)	-0.96 (-2.6 to 0.64)	
	8	Headaches/month	Ryan (1975)	-0.46 (-2.7 to 1.8)	
	8	Headaches/month	Shafar (1972)	-0.72 (-1.6 to 0.14)	
	8	Headaches/month	Stensrud (1976)	-1.0 (-3.1 to 1.1)	
	8		**Pooled (HA/Month):**	**-0.52 (-1.06 to 0.02)**	Q = 1.57, df = 4, I^2^ = 0.0%
	12	Headaches/month	Lynggaard (1975)	-0.37 (-3.4 to 2.)	
	12	Headaches/month	Mondrup (1977)	1.1 (-2.5 to 4.8)	
	12		**Pooled (HA/Month):**	**0.24 (-2.1 to 2.6)**	Q = 0.38, df = 1, I^2^ = 0.0%
	24	Headaches/month	Adam (1978)	0.00 (-0.47 to 0.47)	
**Angiotensin Converting Enzyme Inhibitors**					
Enalapril	4	Headaches/month	Sonbolestan (2013)	-2.4 (-7.5 to 2.7)	
Enalapril	8	Headaches/month	Sonbolestan (2013)	-0.83 (-6.2 to 4.5)	
Lisinopril	12	Headaches/month	Schrader (2001)	-1.4 (-2.6 to -0.2)	
Captopril	16	Headache Index	Paterna (1992)	-0.86 (-1.5 to -0.21)	
**Angiotensin Receptor Blockers**					
Candesaran	12	Headaches/month	Stovner (2013)	-0.58 (-1.4 to 0.23)	
	12	Headaches/month	Trovnik (2003)	-1.6 (-3.0 to -0.16)	
	12		**Pooled (HA/Month)**	**-0.9 (-1.8 to 0.03)**	Q = 1.46, df = 1, I^2^ = 31.7%
Telmisartan	12	Headaches/month	Diener (2009)	-1.9 (-3.6 to -0.23)	—
	12		**Pooled (HA/Month)**	**-1.1 (-1.9 to -0.27)**	**Q = 2.82, df = 3 I** ^**2**^ **= 29.1%**
**Anticonvulsants**					
Acetazolamide	12	Headaches/month	Vahedi (2002)	0.5 (-1.7 to 2.7)	
Carbamazipine	6	Headaches/month	Rompel (1970)	-3.2 (-6.6 to 0.20)	
Carisbamate	14	Headaches/month	Cady (2009)	-0.09 (-0.34 to 0.17)	
Clonazepam	4	Headaches/month	Stensrud (1979)	-3.6 (-7.2 to 0.03))	
Gabapentin	4	Headaches/month	Di’Trapani	-0.2 (-1.6 to 1.2)	
	8	Headaches/month	Di”Trapani	-1.1 (-2.5 to 0.34)	
	12	Headaches/month	Di’ Trapani (2000)	-1.9 (-3.4 to -0.41)	
	12	Headaches/month	Mathew (2001)	-0.2 (-0.89 to 0.49)	
	12		**Pooled (HA/Month)**	—**0.92 (-2.6 to 0.74)**	Q = 4.1, df = 2, I2 = 75.6%
	20	Headaches/month	Silberstein (2013)	-0.33 (-0.71 to 0.04)	
Lamotrigine	4	Headaches/month	Gupta (2006)	-1.2 (-2.2 to -0.18)	
	12	Headaches/month	Steiner (1997)	0.20 (-0.36 to 0.76)	
Levetiracetam	8	Headaches/month	De Tommaso (2007)	-4.2 (-7.2 to -1.3)	
	12	Headaches/month	Verma (2013)	-2.2 (-3.0 to -1.3)	
			**Pooled (HA/Month)**	-2.7 (-4.4 to -0.97)	Q = 1.69, df = 1 I^2^ = 40.9%
Oxcarbazepine	15	Headaches/month	Silberstein (2008)	0.17 (-0.13 to 0.47)	
Topiramate	4 (50 mg/day)	Headaches/month	Brandes (2004)	-0.75 (-1.4 to -0.06)	
	4 (50 mg/day)	Headaches/month	Gupta (2006)	-2.1 (-3.0 to -1.1)	
	4 (50 mg/day)	Headaches/month	Silberstein (2004)	-1.0 (-1.9 to– 0.13)	
	4 (50 mg/day)	Headaches/month	Silvestrini (2003)	-5.0 (-9.2 to -0.8)	
	4 (50 mg/day)		**Pooled (HA/Month)**	**-1.4 (-2.2 to -0.5)**	Q = 7.81, df = 3, I^2^ = 43.0%
Topiramate	4 (100 mg/day)	Headaches/month	Brandes (2004)	-0.80 (-1.4 to -0.18)	
	4 (100 mg/day)	Headaches/month	Diener (2004)	-1.1 (-1.9 to -0.2)	
	4 (100 mg/day)	Headaches/month	Silberstein (2004)	-0.9 (-1.7 to -0.14)	
	4 (100 mg/day)		**Pooled (HA/Month)**	**-0.89 (-1.3 to -0.48)**	Q = 0.23, df = 2, I2 = 0.0%
	4 (200 mg/day)	Headaches/month	Brandes (2004)	-1.9 (-2.4 to -1.3)	
	4 (200 mg/day)	Headaches/month	Edwards (2003)	-0.60 (-1.1 to -0.13)	
	4 (200 mg/day)	Headaches/month	Silberstein (2004)	-1.4 (-2.2 to -0.62)	
	4 (200 mg/day)	Headaches/month	Silberstein (2006)	-0.9 (-2.3 to 0.55)	
	4 (200 mg/day)		**Pooled (HA/Month)**	**-0.91 (-1.3 to -0.48)**	Q = 12.07, df = 3 I2 = 75.1%
	**4 (all doses)**		**Pooled (HA/Month)**	-1.1 (-1.5 to-0.79)	Q = 21.3, df = 10, I^2^ = 53%
	8 (50 mg/day)	Headaches/month	Brandes (2004)	-0.50 (01.2 to 0.19)	
	8 (50 mg/day)	Headaches/month	Silberstein (2004)	-0.80 (-1.4 to -0.20)	
	8 (50 mg/day)	Headaches/month	Silberstein (2006)	-12.5 (-17.1 to -7.9)	
	8 (50 mg/day)	Headaches/month	**Pooled (HA/Month)**	**-2.3 (-4.4 to -0.23)**	Q = 25.56, df = 2, I^2^ = 92.2%
	8 (100 mg/day)	Headaches/month	Brandes (2004)	-0.8 (-1.4 to -0.18)	
	8 (100 mg/day)	Headaches/month	De Tammosa (2007)	-5.2 (-7.8 to -2.5)	
	8 (100 mg/day)	Headaches/month	Diener (2004)	-0.85 (-2.1 to 0.39)	
	8 (100 mg/day)	Headaches/month	Silberstein (2004)	-1.1 (-1.7 to -0.53)	
	8 (100 mg/day)		**Pooled (HA/Month)**	**-1.3 (-2.2 to -0.43)**	Q = 10.18, df = 3, I^2^70.5%
	8 (200 mg/day)	Headaches/month	Brandes (2004)	-1.8 (-2.3 to -1.3)	
	8 (200 mg/day)	Headaches/month	Silberstein (2004)	-1.5 (-2.1 to -0.9)	
	8 (200 mg/day)	Headaches/month	Silberstein (2006)	-0.30 (-1.7 to 1.1)	
	8 (200 mg/day)		**Pooled (HA/Month)**	**-1.5 (-2.1 to -0.87)**	Q = 3.68, df = 2, I^2^ = 45.7%
	8 (All Doses)		**Pooled (HA/Month)**	**-1.3 (-1.9 to -0.7)**	Q = 46.42, df = 9, I^2^ = 80.6%
	12 (50 mg/day)	Headaches/month	Brandes (2004)	-0.40 (-1.1 to 0.29)	
	12 (50 mg/day)	Headaches/month	Silberstein (2004)	-0.70 (-1.4 to 0.02)	
	12 (50 mg/day)		**Pooled (HA/Month):**	**-0.54 (-1.0 to -0.05)**	Q = 0.39, df = 2, I^2^ = 0.0%
	12 (100 mg/day)	Headaches/month	Brandes (2004)	-0.75 (-1.4 to -0.13)	
	12 (100 mg/day)	Headaches/month	Diener (2004)	-0.90 (-2.2 to 0.39)	
	12 (100 mg/day)	Headaches/month	Silberstein (2004)	-1.1 (-2.0 to -0.19)	
	12 (100 mg/day)		**Pooled (HA/Month):**	**-0.87 (-1.3 to -0.39)**	Q = 1.52, df = 2, I^2^ = 0.0%
	12 (200 mg/day)	Headaches/month	Brandes (2004)	-1.7 (-2.2 to -1.1)	
	12 (200 mg/day)	Headaches/month	Silberstein (2004)	-1.4 (-2.2 to -0.59)	
	12 (200 mg/day)	Headaches/month	Silberstein (2006)	-0.70 (-2.1 to 0.75)	
	12 (200 mg/day)		**Pooled (HA/Month):**	**-1.5 (-1.9 to -1.1)**	Q = 0.35, df = 1, I^2^ = 0.0%
	**12 (All doses)**		**Pooled (HA/Month):**	**-0.99 (-1.3 to -0.64)**	Q = 0.04, df = 1, I^2^ = 0.0%
	16 (50 mg/day)	Headaches/month	Brandes (2004)	-0.50 (-1.2 to 0.19)	
	16 (50 mg/day)	Headaches/month	Silberstein (2004)	-0.40 (-1.1 to 0.33)	
	16 (50 mg/day)		**Pooled (HA/Month):**	**-0.45 (-0.95 to 0.05)**	Q = 0.04, df = 1, I^2^ = 0.0%
	16 (100 mg/day)	Headaches/month	Brandes (2004)	-0.70 (-1.3 to -0.08)	
	16 (100 mg/day)	Headaches/month	Diener (2004)	-1.1 (-2.4 to 0.24)	
	16 (100 mg/day)	Headaches/month	Silberstein (2004)	-1.2 (-2.0 to -0.38)	
	16 (100 mg/day)	Headaches/month	Silberstein (2007)	-1.5 (-3.1 to 0.06)	
	16 (100 mg/day)		**Pooled (HA/Month):**	**-0.95 (-1.4 to -0.51)**	Q = 1.51, df = 3, I^2^ = 0.0%
	16 (200 mg/day)	Headaches/month	Brandes (2004)	-1.6 (-2.1 to -1.1)	
	16 (200 mg/day)	Headaches/month	Silberstein (2004)	-1.3 (-2.2 to -0.45)	
	16 (200 mg/day)	Headaches/month	Silberstein (2006)	-0.05 (-1.5 to 1.4)	
	16 (200 mg/day)	Headaches/month	Storey (2001)	-0.52 (-1.67 to 0.63)	
	16 (200 mg/day)		**Pooled (HA/Month):**	**-1.0 (-1.7 to -0.44)**	Q = 5.76, df = 3, I^2^ = 47.9%
	**16 (All doses)**		**Pooled (HA/Month):**	**-0.92 (-1.2 to -0.59)**	Q = 13.41, df = 9, I^2^ = 32.9%
	20 (50mg/day)	Headaches/month	Brandes (2004)	-0.50 (-1.2 to 0.19)	
	20 (50mg/day)	Headaches/month	Silberstein (2004)	-0.55 (-1.5 to 0.40)	
	20 (50mg/day)		**Pooled (HA/Month):**	**-0.52 (-1.1 to 0.04)**	Q = 0.01, df = 1, I^2^ = 0.0%
	20 (100 mg/day)	Headaches/month	Brandes (2004)	-0.70 (-1.3 to -0.08)	
	20 (100 mg/day)	Headaches/month	Diener (2004)	-1.2 (-2.6 to 0.17)	
	20 (100 mg/day)	Headaches/month	Silberstein (2004)	-1.4 (-2.3 to -0.39)	
	20 (100 mg/day)		**Pooled (HA/Month):**	**-0.93 (-1.4 to -0.44)**	Q = 1.42, df = 2, I^2^ = 0.0%
	20 (200 mg/day)	Headaches/month	Brandes (2004)	-1.5 (-2.0 to -0.95)	
	20 (200 mg/day)	Headaches/month	Silberstein (2004)	-1.4 (-2.3 to -0.36)	
	20 (200 mg/day)	Headaches/month	Silberstein (2006)	-0.50 (-1.9 to 0.95)	
	20 (200 mg/day)		**Pooled (HA/Month):**	**-1.4 (-1.8 to 0.92)**	Q = 1.61, df = 2, I^2^ = 0.0%
	20 (all doses)		**Pooled (HA/Month):**	**-0.98 (-1.3 to -0.66)**	Q = 8.52, df = 7, I^2^ = 17.8%
	24 (50 mgday)	Headaches/month	Brandes (2004)	-0.40 (-1.2 to 0.44)	
	24 (50 mgday)	Headaches/month	Silberstein (2004)	-0.50 (-1.5 to 0.48)	
	24 (50 mgday)		**Pooled (HA/Month):**	**-0.44 (-1.1 to 0.20)**	Q = 0.02, df = 1, I^2^ = 0.0%
	24 (100 mg/day)	Headaches/month	Brandes (2004)	-1.0 (-1.8 to -0.18)	
	24 (100 mg/day)	Headaches/month	Lipton (2011)	-1.4 (-2.2 to -0.60)	
	24 (100 mg/day)	Headaches/month	Silberstein (2004)	-1.3 (-2.3 to -0.34)	
	24 (100 mg/day)		**Pooled (HA/Month):**	**-1.2 (-1.7 to -0.74)**	Q = 0.49, df = 2, I^2^ = 0.0%
	24 (200 mg/day)	Headaches/month	Brandes (2004)	-1.5 (-2.2 to -0.84)	
	24 (200 mg/day)	Headaches/month	Silberstein (2004)	-1.3 (-2.3 to -0.31)	
	24 (200 mg/day)		**Pooled (HA/Month):**	**-1.4 (-2.0 to -0.89)**	Q = 0.11, df = 1, I^2^ = 0.0%
	**24 (All doses)**		**Pooled (HA/Month):**	**-1.1 (-1.4 to -0.77)**	Q = 6.4, df = 6, I^2^ = 6.4%
Valproate	4	Headaches/month	Freitag (2002)	-0.20 (-0.61 to 0.21)	
	4	Headaches/month	Klapper (1997)	-1.8 (-2.6 to -0.95)	
	4	Headaches/month	Mathew (1995	-1.8 (-3.6 to -0.03)	
	4		**Pooled (HA/Month):**	**-1.4 (-2.2 to -0.56)**	Q = 14.48, df = 2, I^2^ = 51.6%
	8	Headaches/month	Freitag (2002)	-0.25 (-0.51 to 0.01)	
	8	Headaches/month	Hering (1992)	-6.8 (-12.10 to -1.5)	
	8	Headaches/month	Klapper (1997)	-1.6 (-2.3 to -0.95)	
	8	Headaches/month	Mathew (1995)	-2.1 (-3.6 to -0.56)	
	8		**Pooled (HA/Month):**	**-1.5 (-2.2 to -0.76)**	Q = 17.35, df = 3. I^2^ = 42.6%
	12	Headaches/month	Freitag (2002)	-0.45 (-0.86 to -0.04)	
	12	Headaches/month	Jensen (1994)	-2.6 (-5.5 to 0.26)	
	12	Headaches/month	Klapper (1997)	-1.7 (-2.4 to -0.96)	
	12	Headaches/month	Mathew (1995)	-2.8 (-4.8 to -0.74)	
	12		**Pooled (HA/Month):**	**-1.5 (-2.1 to -0.80)**	Q = 24.7, I^2^ = 63.6%
Vigabatrin	4	Headaches/month	Ghose (2002)	-0.54 (-1.9 to 0.77)	
	8	Headaches/month	Ghose (2002)	-0.27 (-2.3 to 1.7)	
	12	Headaches/month	Ghose (2002)	-0.42 (-2.3 to 1.6)	
**Beta-Blockers**					
Acebutolol	4	Headaches/month	Nanda (1977)	0.10 (-0.75 to 0.95)	
	8	Headaches/month	Nanda (1977)	-0.50 (-1.35 to 0.35)	
	12	Headaches/month	Nanda (1977)	-0.68 (-1.68 to 0.32)	
Alprenolol	8	Headaches/month	Ekbom (1975)	0.20 (-0.91 to 1.3)	
Atenolol	8	Headaches/month	Stensrud (1980)	-1.5 (-3.0 to -0.04)	
	12	Headaches/month	Forssman (1983)	-5.4 (-12.6 to 1.8)	
	12	Headaches/month	Johansson (1987)	-2.05 (-3.76to -0.48)	
	12		**Pooled SMD**	**-2.2 (-3.7 to -0.67)**	Q = 0.80, df = 1, I^2^ = 0.0%
Bisoprolol	4	Headaches/month	Van de Ven (1997)	-0.40 (-0.87 to 0.07)	
	8	Headaches/month	Van de Ven (1997)	-0.61 (-1.1 to -0.16)	
Metoprolol	4	Headaches/month	Langor (1985)	-0.63 (-1.5 to 0.25)	
	8	Headaches/month	Andersson (1983)	-1.5 (-2.4 to -0.60)	
	8	Headaches/month	Kangasniemi (1987)	-0.70 (-1.4 to -0.03)	
	8	Headaches/month	Steiner (1987)	-0.80 (-1.7 to 0.13)	
	8		**Pooled HA/Month**	**-0.94 (-1.4 to -0.46)**	Q = 1.02, I^2^ = 0.0%
Oxprenolol	8	Headaches/month	Ekbom (1977)	-080 (-3.9 to 2.3)	
Pindolol	4	Headaches/month	Ekbom (1972)	2.9 (-1.0 to 6.8)	
	4	Headaches/month	Sjaastad (1972)	1.5 (-5.5 to 2.6)	
	4		**Pooled HA/Month**	**1.2 (-2.5 to 4.9)**	Q = 0.49, I^2^ = 0.0%
Propranolol	4	Headaches/month	Dahlof (1987)	-1.1 (-2.2 to 0.05)	
	4	Headaches/month	Diener (2004)	-1.1 (-1.9 to -0.28)	
	4	Headaches/month	Pradalier (1989)	-1.5 (-2.1 to -0.87)	
	4	Headaches/month	Stensrud (1976)	-1.1 (-3.5 to 1.3)	
	4		**Pooled HA/Month**	**-1.1 (-1.5 to -0.74)**	Q = 3.44, df = 3, I^2^ = 0.00%
	4	Headache Index	Zeigler (1987)	-0.68 (-1.4 to 0.06)	
	8	Headaches/month	Ahuja (1985)	-5.9 (-11.4 to -0.37)	
	8	Headaches/month	Al-Qassab (1993)	0.6 (-2.84 to 4.0)	
	8	Headaches/month	Diener (2004)	-0.8 (-1.6 to -0.005)	
	8	Headaches/month	Holroyd (2010)	-0.6 (-1.3 to 0.12)	
	8	Headaches/month	Pita (1977)	-5.3 (-8.7 to -1.8)	
	8		**Pooled HA/Month**	**-1.0 (-2.1 to -0.39)**	Q = 11.08, df = 4, I^2^ = 54.9%
	8	Headache index	Nadelmann (1986)	-0.54 (-1.11 to 0.04)	
	12	Headaches/month	Borgesen (1974)	-0.30 (-1.1 to -0.48)	
	12	Headaches/month	Diener (2004)	-0.80 (-1.6 to -0.02)	
	12	Headaches/month	Forssman (1976)	-1.8 (-3.9 to 0.4)	
	12	Headaches/month	Holroyd (2010)	-0.8 (-1.5 to -0.08)	
	12	Headaches/month	Johnson (1986)	-6.3 (-16.6 to 3.9)	
	12	Headaches/month	Mikkelsen (1986)	-2.4 (-7.9 to 3.1)	
	12	Headaches/month	Pradalier (1989)	-3.3 (-3.9 to -2.6)	
	12	Headaches/month	Standnes (1982)	-1.4 (-2.7 to -0.12)	
	12	Headaches/month	Stovner (2013)	-0.62 (-1.4 to 0.16)	
	12	Headaches/month	Tfelt-Hansen (1983)	-1.2 (-2.8 to 0.45)	
	12	Headaches/month	Wideroe (1974)	-1.3 (-1.8 to -0.78)	
	12		**Pooled HA/Month**	**-1.3 (-2.0 to -0.62)**	Q = 46.35, df = 10, I^2^ = 78.4%
	12	Headache hours/month	Diener (1996)	-12.9 (-31.8 to 5.9)	
	16	Headaches/month	Diener (2004)	-1.1 (-2.2 to -0.01)	
	16	Headaches/month	Holroyd (2010)	-0.90 (-2.6 to -0.19)	
	16	Headaches/month	Sargent (1985)	0.5 (-0.22 to 1.2)	
			**Pooled HA/Month**	**-0.46 (-1.5 to 0.57)**	Q = 9.40, df = 2, I^2^ = 78.7%
	20	Headaches/month	Diener (2004)	-1.5(-2.9 to -0.03)	
	20	Headaches/month	Holroyd (2010)	-0.9 (-1.6 to -0.18)	
	20		**Pooled HA/Month**	**-1.0 (-1.7 to -0.37)**	Q = 0.52, df = 1, I^2^ = 0.0%
Tomolol	8	Headaches/month	Briggs (1979	-2.3 (-5.2 to 0.63)	
	8	Headaches/month	Stellar (1984)	-0.70 (-1.5 to 0.07)	
	8		**Pooled HA/Month**	**-0.85 (-1.8 to 0.07)**	Q = 1.01, df = 1, I^2^ = 6.8%
	12	Headaches/month	Standnes (1982)	-1.9 (-3.2 to -0.54)	
	12	Headaches/month	Tfelt-Hansen (1984)	-1.5 (-3.0 to 0.05)	
	12	Headaches/month	**Pooled HA/Month**	**-1.7 (-2.7 to -0.70)**	Q = 0.12, df = 1, I^2^ = 0.0%
**Calcium Channel Blockers**					
Cyclendelate	12	Headache duration/month (hours)	Diener (1996)	15.0 hours (-5.3 to 35.3)	
Nicardipine	8	Headaches/month	Leandri (1990)	-1.6 (-3.3 to 0.15)	
Nifedipine	4	Headaches/month	McArthur (1989)	-0.20 (-0.72 to 0.32)	
	4	Headaches/month	Shukla (1995)	-3.8 (-4.8 to -2.8)	
	4		**Pooled HA/Month**	**-2.0 (-5.5 to 1.6)**	Q = 2.92, df = 1, I^2^ = 65.8%
	8	Headaches/month	McArthur (1989)	-0.20 (-0.72 to 0.32)	
	12	Headaches/month	McArthur (1989)	-0.40 (-1.4 to 0.66)	
Nimodipine	4	Headaches/month	Gelmers (1983)	-0.13 (-0.64 to 0.38)	
	4	Headaches/month	MINES (1989)	0.48 (-0.47 to 0.46)	
	4	Headaches/month	MINES (1989)	0.10 (-0.21 to 0.41)	
	4	Headaches/month	Stewart (1988)	-0.31 (-3.3 to 2.7)	
	4		**Pooled HA/Month**	**-0.07 (-0.28 to 0.13)**	Q = 1.51, df = 3, I^2^ = 0.0%
	4	Headache Index	Ansell (1988)	-0.36 (-0.88 to 0.16)	
	8	Headaches/month	Gelmers (1983)	-1.5 (-2.4 to -0.73)	
	8	Headaches/month	Havanka (1985)	-2.2 (-4.2 to -0.24)	
	8	Headaches/month	MINES (1989)	0.17 (-0.54 to 0.88)	
	8	Headaches/month	MINES (1989)	0.94 (-0.45 to 2.32)	
	8	Headaches/month	Stewart (1988)	-3.4 (-5.7 to -1.1)	
	8		**Pooled HA/Month**	**-0.98 (-2.3 to 0.30)**	Q = 22.35, df = 4, I^2^ = 82.1%
	8	Headache Index	Ansell (1988)	-0.48 (-1.01 to 0.05)	
	12	Headaches/month	Gelmers (1983)	-1.3 (-1.9 to -0.69)	
	12	Headaches/month	MINES (1989)	0.74 (0.03 to 1.5)	
	12	Headaches/month	MINES (1989)	-0.01 (-1.4 to 1.4)	
	12	Headaches/month	Stewart (1988)	-2.8 (-5.2 to -0.48)	
	12		**Pooled HA/Month**	**-0.65 (-2.0 to 0.74)**	Q = 22.41,df-, I^2^ = 86.6%
	12	Headache Index	Ansell (1988)	0.16 (-0.36 to 0.68)	
Verapamil	4	Headaches/month	Solomon (1983)	-2.9 (-7.8 to 1.9)	
	8	Headaches/month	Markley (1984)	-0.60 (-1.4 to 0.19)	
**Flunarizine**					
Flunarizine	4	Headaches/month	Diamond (1993)	0.60 (-0.35 to 0.47)	
	4	Headaches/month	Frenken (1984)	-1.3 (-2.4 to -0.24)	
	4		**Pooled Ha/Month**	**-0.53 (-1.8 to 0.79)**	Q = 5.51, df = 1, I^2^ = 81.9%
	4	Headache Index	Mendenopoulos (1985)	-0.63 (1.5 to 0.27)	
	4	Headache Index	Pini (1986)	0.19 (-0.73 to 1.1)	
	4	Headache Index	**Pooled SMD**	**-0.23 (-1.0 to 0.58)**	Q = 1.56, df = 1, I^2^ = 35.8%
	4		**Overall Pooled SMD**	**-0.27 (-0.76 to 0.23)**	Q = 6.15, df = 3, I^2^ = 51.2%
	8	Headaches/month	Diamond (1993)	-0.44 (-1.7 to 0.78)	
	8	Headaches/month	Frenken (1984)	-1.9 (-3.5 to -0.31)	
	8		**Pooled Ha/Month**	**-1.1 (-2.5 to 0.34)**	Q = 2.04, df = 1, I^2^ = 51.1%
	8	Headache Index	Mendenopoulos (1985)	-1.2 (-2.1 to -0.2)	
	8		**Overall Pooled SMD**	**-0.60 (-1.2 to 0.005)**	Q = 4.92, df = 2, I^2^ = 59.3%
	12	Headaches/month	Diamond (1993)	-0.61 (-1.8 to 0.65)	
	12	Headaches/month	Frenken (1984)	-1.8 (-3.3 to -0.38)	
	12	Headaches/month	Louis (1981)	-1.1 (-1.7 to -0.6)	
	12		**Pooled Ha/Month**	**-1.1 (-1.6 to -0.67)**	Q = 1.39, df = 2, I^2^ = 0.0%
	12	Headache Index	Mendenopoulos (1985)	-1.6 (-2.6 to -0.6)	
	12	Headache Index	Thomas (1989)	-0.87 (2.0 to 0.24)	
	12	Headache Index	**Pooled SMD**	**-1.3 (-2.0 to -0.52)**	Q = 0.94, df = 1, I^2^ = 0.0%
	12		**Overall Pooled SMD**	**-0.84 (-1.3 to -0.34)**	Q = 10.33, df = 4, I^2^ = 61.3%
	16	Headaches/month	Diamond (1993)	-1.2 (-2.1 to -0.24)	
	16	Headaches/month	Sorensen (1986)	-1.2 (-2.7 to 0.37)	
	16		**Pooled Ha/Month**	**-1.2 (-2.0 to -0.38)**	
	20	Headaches/month	Diamond (1993)	-0.36 (-2.4 to 1.69)	
**Selective Serotonin Reuptake Inhibitors**					
	4	Headaches/month	Orholm (1986)	-0.20 (-1.3 to 0.93)	
	4	Headaches/month	Zeeberg (1981	0.00 (-1.8 to 1.8)	
Femoxitine	4		**Pooled Ha/Month**	**-0.14 (-1.1 to 0.81)**	Q = 0.03, df = 1, I^2^ = 0.0%
	8	Headaches/month	Orholm (1986)	-0.10 (-1.2 to 1.0)	
	8	Headaches/month	Zeeberg (1981)	-1.8 (-3.6 to -0.04)	
	8		**Pooled Ha/Month**	—**0.81 (-2.5 to 0.83)**	Q = 2.53, df-1, I^2^ = 60.4%
	12	Headaches/month	Orholm (1986)	0.50 (-0.63 to 1.6)	
	12	Headaches/month	Zeeberg (1981)	-1.4 (-3.2 to 0.36)	
	12		**Pooled Ha/Month**	**-0.33 (-2.2 to 1.5)**	Q = 3.16, df = 1, I^2^ = 68.3%
	16	Headaches/month	Orholm (1986)	0.30 (-0.83 to 1.4)	
Fluoxetine	4	Headache index	Adly (1993)	-0.34 (-1.27 to 0.59)	
	4	Headache index	d’Amato (1999)	-0.08 (-0.63 to 0.48)	
	4	Headaches/month	Steiner (1998)	-0.71 (-1.36 to -0.06)	
	4		**Pooled SMD**	**-0.35 (-0.75 to 0.05)**	Q = 2.11, df = 3, I^2^ = 5.2%
	8	Headache index	Adly (1993)	-0.74 (-1.70 to 0.22)	
	8	Headache index	d’Amato (1999)	-0.01 (-0.56 to 0.55)	
	8	Headaches/month	Steiner (1998)	-0.32 (-0.98 to 0.35)	
	8		**Pooled SMD**	**-0.23 (-0.63 to 0.16)**	Q = 1.76, I^2^ = 0.0%
	12	Headache index	Adly (1993)	-1.02 (-2.01 to -0.03)	
	12	Headache index	d’Amato (1999)	-0.32 (-0.88 to 0.24)	
	12	Headaches/month	Steiner (1998)	-0.74 (-1.44 to -0.03)	
	12		**Pooled SMD**	**-0.57 (-0.97 to -0.17)**	Q = 1.77, df = 2, I^2^ = 0.0%
	16	Headache index	d’Amato (1999)	-0.64 (-1.22 to -0.07)	
	20	Headache index	d’Amato (1999)	-0.32 (-0.88 to 0.24)	
Sertraline	4	Headache index	Landy (1999)	0.44 (-0.59 to 1.5)	
	8	Headache index	Landy (1988)	0.08 (-0.94 to 1.09)	
**Pooled SMD (12 weeks)**				**-0.35 (-0.75 to 0.05)**	Q = 7.49, df = 4, I^22^ = 46.6%
**Serotonin Norepinephrine Reuptake Inhibitors**					
Venlafaxine	8	Headaches/month	Ozyalcin (2004)	-2.0 (-4.0 to -0.06)	
**Serotonin Antagonist**					
Pizotifen	4	Headaches/month	Arthur (1971)	-0.51 (-1.07 to 0.05)	
	4	Headaches/month	Ryan (1968)	-0.36 (-0.86 to 0.15)	
	4		**Pooled HA/month**	**-0.42 (-0.80 to -0.05)**	Q = 0.16m df = 1, I^2^ = 0.0%
	4	Headache index	Carroll (1975)	0.18 (-0.87 to 1.23)	
	4	Headache index	Lawrence (1977)	-0.04 (-0.78 to 0.70)	
	4		**Pooled SMD**	**-0.15 (-0.47 to 0.17)**	Q = 6.40, df = 1, I^2^ = 0.0%
	4		**Overall Pooled SMD**	**-0.30 (-0.62 to 0.02)**	Q = 6.40, df = 7, I^2^ = 0.0%
	8	Headaches/month	Kangasniemi (1979)	-0.57 (-1.26 to 0.12)	
	8	Headaches/month	Osterman (1977)	-0.63 (-1.4 to 0.1)	
	8		**Pooled HA/month**	**-0.60 (-1.1 to -0.08)**	Q = 0.01, df = 1, I^2^ = 0.0%
	8	Headache index	Lawrence (1977)	-0.56 (-1.32 to 0.20)	
	8		**Overall Pooled SMD**	**-0.48 (-0.85 to -0.12)**	Q = 1.37, df = 4, I^2^ = 0.0%
	12	Headaches/month	Bellavance (1990)	-0.49 (-0.86 to -0.12)	
	12	Headaches/month	Cleland (1997)	-0.41 (-0.83 to 0.14)	
	12	Headaches/month	Hughes (1971)	-0.26 (-1.03 to 0.52)	
	12		**Pooled HA/month**	**-0.43 (-0.66 to -0.21)**	Q = 0.30, df = 2, I^2^ = 0.0%
	12	Headache index	Lawrence (1977)	-0.56 (-1.32 to 0.20)	
			**Overall Pooled SMD**	**-0.44 (-0.69 to -0.20)**	Q = 0.48, df = 5, I^2^ = 0.0%
**Tricyclic Antidepressants**					
Amitriptyline	4	Headache index	Couch (2011)	-0.34 (-0.62 to -0.05)	
	4	Headache index	Zeigler (1987)	-0.52 (-1.25 to 0.21)	
	4		**Pooled SMD**	**-0.57 (-0.92 to -0.23)**	Q = 0.08, df = 1, I^2^ = 0.0%
	24	Headaches/month	Gomersall (1973)	-1.29 (-1.79 to -0.46)	
	24	Headache index	Mathew (1981)	-1.31 (-1.85 to -0.77)	
	24		**Pooled SMD**	**-1.2 (-1.7 to -0.82)**	Q = 0.35, df = 2, I^2^ = 0.0%
Clomipramine	4	Headaches/month	Langohr (1985)	0.10 (-1.2 to 1.01)	
	4	Headaches/month	Loldrup (1989)	-0.51 (-0.81 to -0.20)	
	4	Headaches/month	Noone (1980)	-0.3 (-1.19 to 0.58)	
	4		**Pooled SMD**	**-0.46 (-0.74 to -0.18)**	
Doxepin	4	Headache index	Morland (1979)	-0.77 (-1.54 to 0.00)	—
Opipramol	4	Headaches/month	Jacobs (1972)	-1.2 (-2.1 to -0.3)	
	12		Jacobs (1972)	-1.3 (-2.5 to -0.12)	

SMD: Standardized Mean Difference

**Table 6:** Placebo controlled comparisons of continuous outcomes among patients with chronic migraine headache (≥ 15 headaches/month).

**Chronic Daily Headache**					
Fluoxetine	12	Headaches/month	Saper (1994)	-0.40 (-1.1 to 0.35)	—
Gabapentin	8	Headaches/month	Spira (2003)	-2.7 (-5.2 to 0.28)	—
Levetiracetam	82	Headaches/month	Beran (2010)	-3.6 (-7.7 to 0.56)	—
**Chronic Migraines (15 or more headaches/month)**					
Atenolol	6	Headaches/month	Stensrud (1980)	0.32 (-0.73 to 1.38)	—
Propranolol	8	Headaches/month	Palferman (1988)	-0.70 (-1.3 to -0.09)	
	6	Headaches/month	Stensrud (1980)	0.24 (-0.82 to 1.29)	
			**Pooled SMD**	**-0.34 (-1.23 to 0.56)**	Q = 0.13, df = 2, I^2^ = 56.1%
Tizanidine	4	Headaches/month	Saper (2002)	-1.1 (—2.4 to 0.16)	—
	8	Headaches/month	Saper (2002)	-1.0 (-2.3 to 0.30)	—
	12	Headaches/month	Saper (2002)	-0.50 (-1.6 to 0.62)	—
Topiramate	4	Headaches/month	Diener (2007)	-4.9 (-7.7 to -2.1)	
	4	Headaches/month	Mei (2006)	-9.2 (-15.7 to -2.7)	
	4	Headaches/month	Silvestrini (2003)	-5.0 (-9.2 to -0.81)	
	4		**Pooled (HA/Month)**	**-5.4 (-7.6 to -3.2)**	Q = 0.34, df = 2, I^2^ = 0.0%
	8	Headaches/month	Diener (2007)	-3.1 (-5.9 to -0.29)	
	8	Headaches/month	Mei (2006)	-12.7 (-10.2 to -6.2)	
	8	Headaches/month	Silvestrini (2003)	-12.5 (-17.1 to -7.9)	
			**Pooled (HA/Month):**	**-9.1 (-16.3 to -1.9)**	Q = 9.33, df = 2, I^2^ = 78.6%
	12	Headaches/month	Diener (2007)	-6.0 (-8.8 to -3.2)	
	12	Headaches/month	Mei (2006)	-12.2 (-18.7 to -5.7)	
			**Pooled (HA/Month):**	**-8.4 (-14.3 to -2.5)**	Q = 0.81, df = 1, I^2^ = 0.0%
	16		Diener (2007)	-7.6 (-10.4 to -4.8)	
Valproate	4	Headaches/month	Yurekli (2008)	-12.6 (-17.9 to -7.3)	
	12	Headaches/month	Sarchelli (2014)	-4.3 (-7.1 to -1.5)	
	12	Headaches/month	Yurekli (2008)	-14.3 (-19.5 to -9.1)	
	12		**Pooled (HA/Month):**	**-10.9 (-18.5 to -3.4)**	Q = 26.2, df = 1, I^2^ = 92.4%

#### Angiotensin Converting Enzyme Inhibitors (ACE)/ Angiotensin Receptor Blockers (ARB)

**Fig 3:**
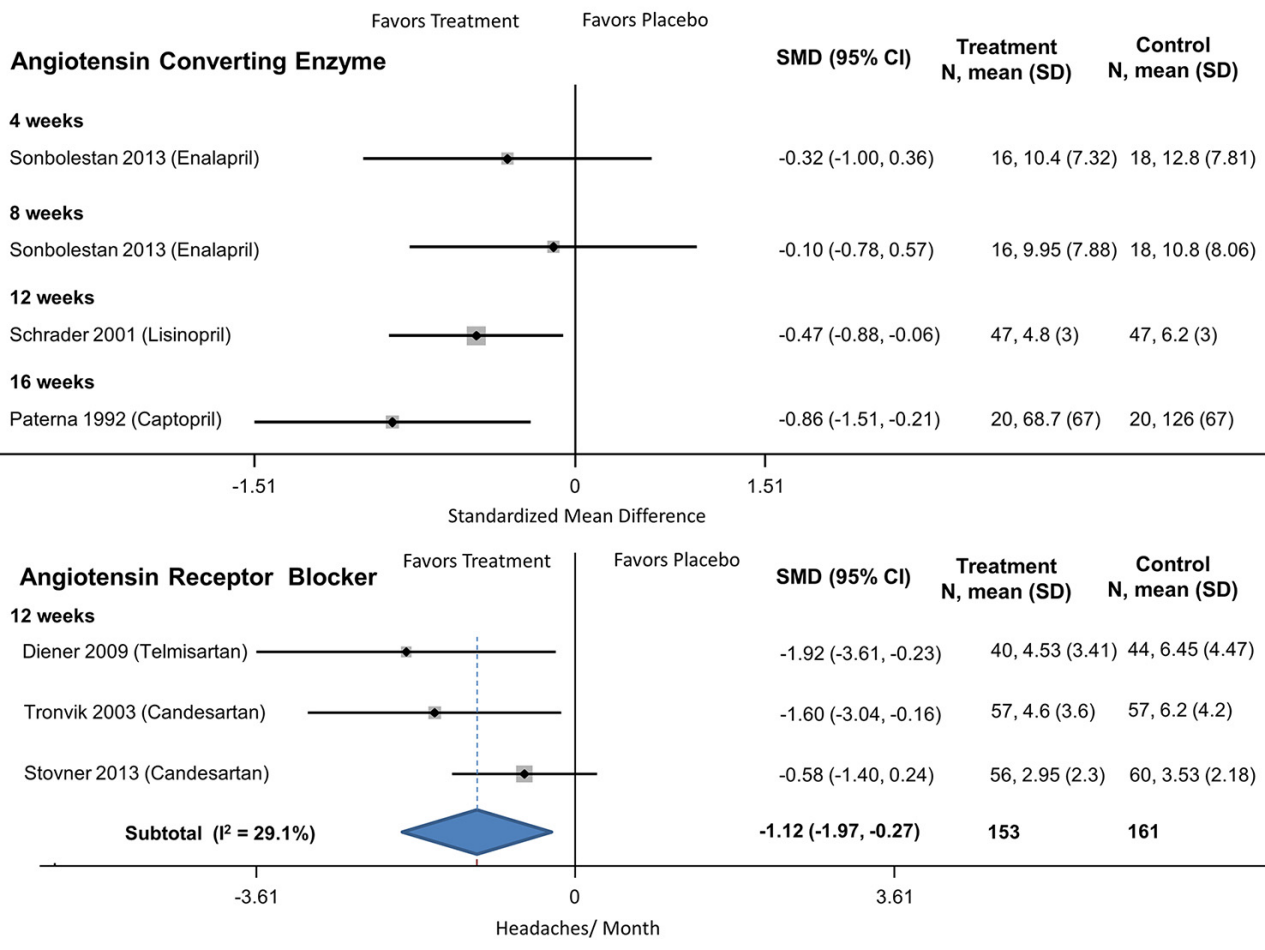
ACE and ARBs compared to placebo for episodic migraine headaches.

There were three ACE (captopril, enalapril, lisinopril) and three ARB (candesartan x2, telmisartan) placebo-controlled trials, all focusing on episodic migraines (Table 2). The ACE studies were 8, 12 and 16 weeks in duration with 120 participants who averaged 7.3 headaches per month. All three ARB studies were 12 weeks in duration with a total of 231 participants, averaging 6.5 headaches/month. One of the ACE trials suggested no benefit at 4 or 8 weeks (enalapril), another found benefit at 12 weeks (lisinopril) and a third benefit at 16 weeks (captopril, Table 5, Fig 3); none of the trials reported outcomes at a common time-point. At twelve weeks, ARBs were better than placebo in reducing the frequency of headaches (Table 5, Fig 3). The likelihood of experiencing at least 50% improvement was not reported in all clinical trials. One of the ACE trials (captopril) was more likely than placebo to achieve at least a 50% reduction in headache frequency (Table 7). This was not found in the trial studying lisinopril or for two of the ARB trials.

**Table 7:** Placebo controlled comparisons of >50% improvement in episodic migraine headaches (<15 migraines/month).

Drug	Time Point (weeks)	Study (Year)	RR (95% CI)	Heterogeneity
**Angiotensin Converting Enzyme Inhibitors**				
Captopril	8	Sonbolestan (2013)	5.6 (1.4–21.9)	
Lisinopril	12	Schrader (2012)	0.82 (0.46–1.5)	
**Angiotensin Receptor Blockers**				
Candesartan	12	Tronvik (2003)	18.0 (2.5–130.4)	
Telmisartan	12	Diemer (2009)	1.6 (0.85–3.0)	
	12	**Pooled RR**	**4.4 (0.43–46.2)**	Q = 5.2, df = 1 I^2^ = 80.8%
**Anticonvulsants**				
Acetazolamide	12	Vahedi (2002)	0.92 (0.42–2.0)	
Carisbamate	12	Cady (2009)	0.75 (0.58–0.98)	
Lamotrigine	4	Gupta (2006)	1.4 (0.86–2.2)	
	12	Steiner (1997)	0.20 (-0.36 to 0.76)	
Levetiracetam	12	Verma (2003)	1.4 (0.86–2.4)	
Oxcarbazepine	15	Silberstein (2008)	0.90 (0.59–1.4)	
Topiramate	4	Edwards (2003)	4.2 (1.3–13.7)	
	4	Gupta (2006)	2.1 (1.3–3.2)	
	4	**Pooled RR:**	**2.4 (1.3–4.2)**	Q = 11.27, df = 1, I^2^ = 21.0%
	12	Silberstein (2006)	1.2 (0.8–1.7)	
	16	Silberstein (2004)	2.1 (1.6–2.7)	
	16	Silberstein (2009)	1.3 (0.9–2.0)	
	16	**Pooled RR:**	**1.9 (1.4–2.5)**	Q = 6.4, df = 1, I^2^ = 52.9%
	26	Brandes (2004)	1.7 (1.3–2.2)	
	26	Diener (2004)	1.6 (1.1–2.4)	
	26	**Pooled RR:**	**1.8 (1.5–2.2)**	Q = 1.72, df = 1, I^2^ = 0.0%
	26	Freitag (2002)	1.2 (0.8–1.9)	
Valproate	12	Jensen (1994)	2.8 (1.3–6.3)	
	12	Klapper (1997)	2.3 (1.6–3.3)	
	12	Mathew (1995)	3.6 (1.5–8.4)	
	12	**Pooled RR**	**2.1 (1.5–3.0)**	Q = 9.1, df = 3, I^2^ = 45.1%
**Beta-blockers**				
Propranolol	4	Stensrud (1976)	1.25 (0.55–2.8)	
	8	Pita (1977)	17.0 (1.0–281.9)	
	8	Zeigler (1993)	2.5 (0.65–9.7)	
	8	**Pooled RR:**	**4.3 (0.79–23.6)**	Q = 1.45, df = 1, I^2^ = 31.1%
	12	Telt-Hansen (1984)	2.0 (1.2–2.8)	
	12	Weber (1972)	7.5 (1.9–28.4)	
	12	Wideroe (1974)	2.2 (1.4–3.4)	
	12	**Pooled RR:**	**2.1 (1.6–2.9)**	Q = 4.2, df = 2, I^2^ = 52.2%
	24	Diener (1996)	1.4 (0.9–2.2)	
	26	Diener (2004)	2.0 (1.4–2.9)	
Metoprolol	4	Langohr (1985)	1.2 (0.86–1.5)	
Timolol	8	Stellar (1984)	1.6 (1.1–2.4)	
	12	Tfelt-Hansen (1984)	1.9 (1.4–2.5)	
**Calcium Channel Blockers**				
Cinnarizine	4	Togha (2007)	0.98 (0.74–1.3)	
Cyclendalate	24	Diener (1996)	1.3 (0.8–2.1)	
Flunarizine	12	Thomas (1991)	2.5 (0.6–10.9)	
	16	Bunoso (1998)	0.99 (0.72–1.4)	
	16	Diener (2002)	1.0 (0.88–1.2)	
	16	**Pooled RR:**	**1.02 (00.91–1.1)**	Q = 1.6, df = 1, I^2^ = 82.4%
Nifedipine	24	Albers (1989)	0.45 (0.21–0.95)	
Fluoxetine	4	Singh (2002)	4.5 (1.1–18.8)	
	12	Saper (1994)	1.0 (0.57–1.8)	
**Tricyclic Antidepressants**				
Amitriptyline	4	Couch (1976)	2.2 (1.0–4.8)	
	4	Couch (1979)	1.60 (1.0–2.5)	
	4	**Pooled RR:**	**1.7 (1.2–2.6)**	Q = 0.54,df = 1, I^2^ = 0.0%
	8	Nelson (1998)	2.22 (1.3–3.9)	
	8	Zeigler (1993)	0.83 (0.43–1.6)	
	8	**Pooled RR:**	**1.1 (0.6–2.0)**	Q = 0.64,df = 1, I^2^ = 3.0%
	12	Canepari (1985))	1.60 (0.31–3.1)	
	26	Dodick (2009)	0.82 (0.61–1.1)	
Clomipramine	4	Langohr (1985)	0.94 (0.53–1.7)	
**Tetracyclic**				
Maprotiline	12	Amelin (2000)	0.76 (0.32–1.8)	

#### Anticonvulsants

There were 32 trials comparing anticonvulsants to placebo with a total of 8529 participants who averaged 41 years (range 12–76) in age; 81% of participants were women (Table 2). Twenty-seven of these trials focused on episodic migraine headaches (Table 2), five evaluated chronic migraine and four chronic daily headaches (Table 3). The average rate of withdrawals was 23%. Studies averaged 15 weeks (range 4–82) with a mean of 153 participants (range 23–487). All of the studies reported headache frequency as their outcome. The two most commonly tested anticonvulsants were topiramate (n = 12) and valproate (n = 6). Other anticonvulsants tested included acetazolamide (n = 1), carbamazepine (n = 1), carisbamate (n = 1), clonazepam (n = 1), gabapentin (n = 4), lamotrigine (n = 1), levetiracetam (n = 3), oxcarbazepine (n = 1), and vigabatrin (n = 1).

In single trials, several anticonvulsants were no better than placebo for episodic migraines including acetazolamide, carbamazepine, carisbamate, clonazepam, oxcarbazepine and vigabatrin (Table 5). In single trials, lamotrigine was found effective at 4 weeks though ineffective at 12 weeks (Table 5). In several trials, gabapentin was not superior to placebo (Table 5). Several of these anticonvulsants were assessed for ability to reduce headaches by 50% (Table 7). Carisbamate was less effective than placebo and anticonvulsants no more likely than placebo to reduce headaches by at least 50% included acetazolamide, gabapentin, lamotrigine, levetiracetam and oxcarbazepine.

**Fig 4:**
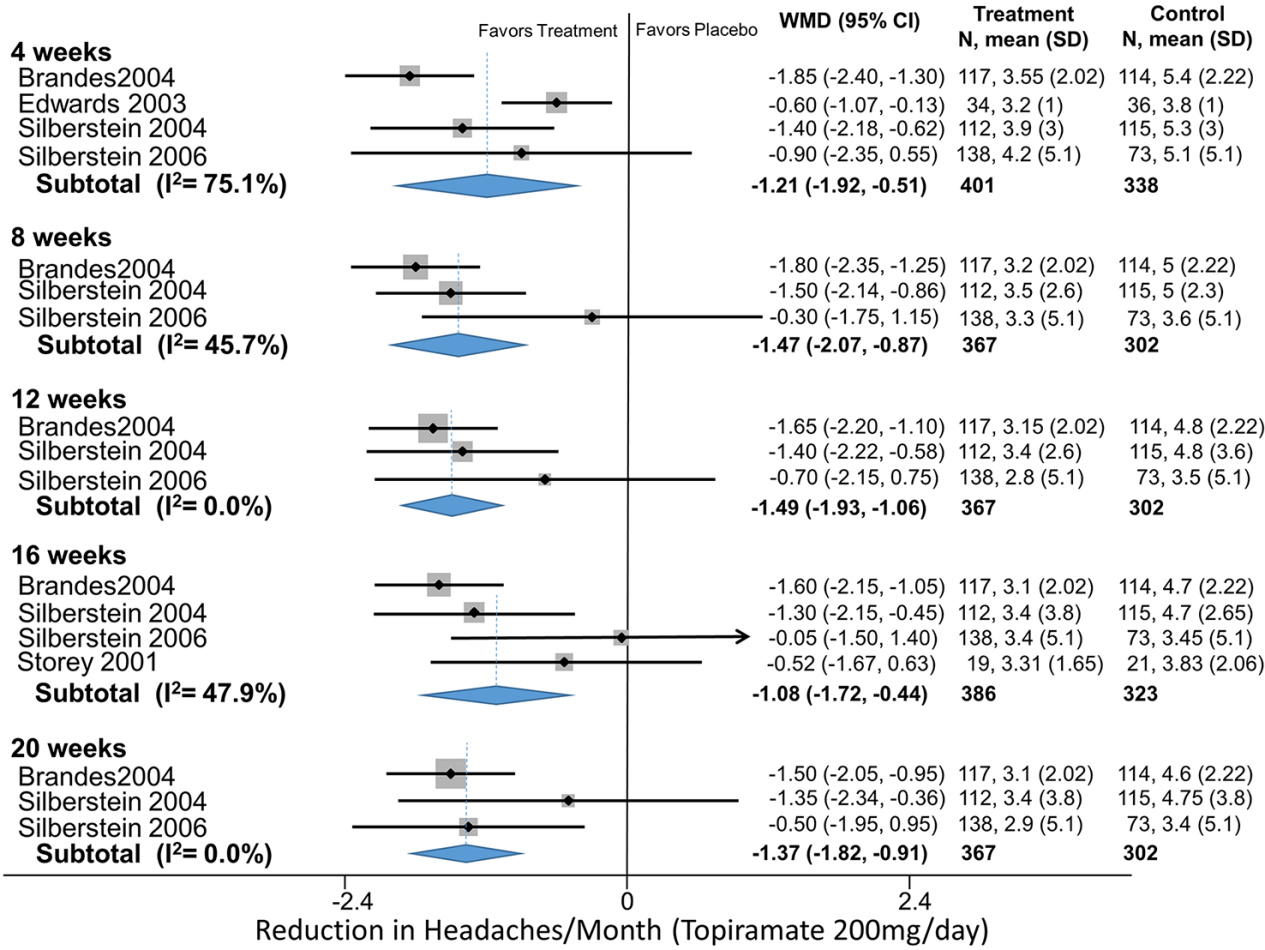
Topiramate compared to placebo for episodic migraine headaches.

**Fig 5:**
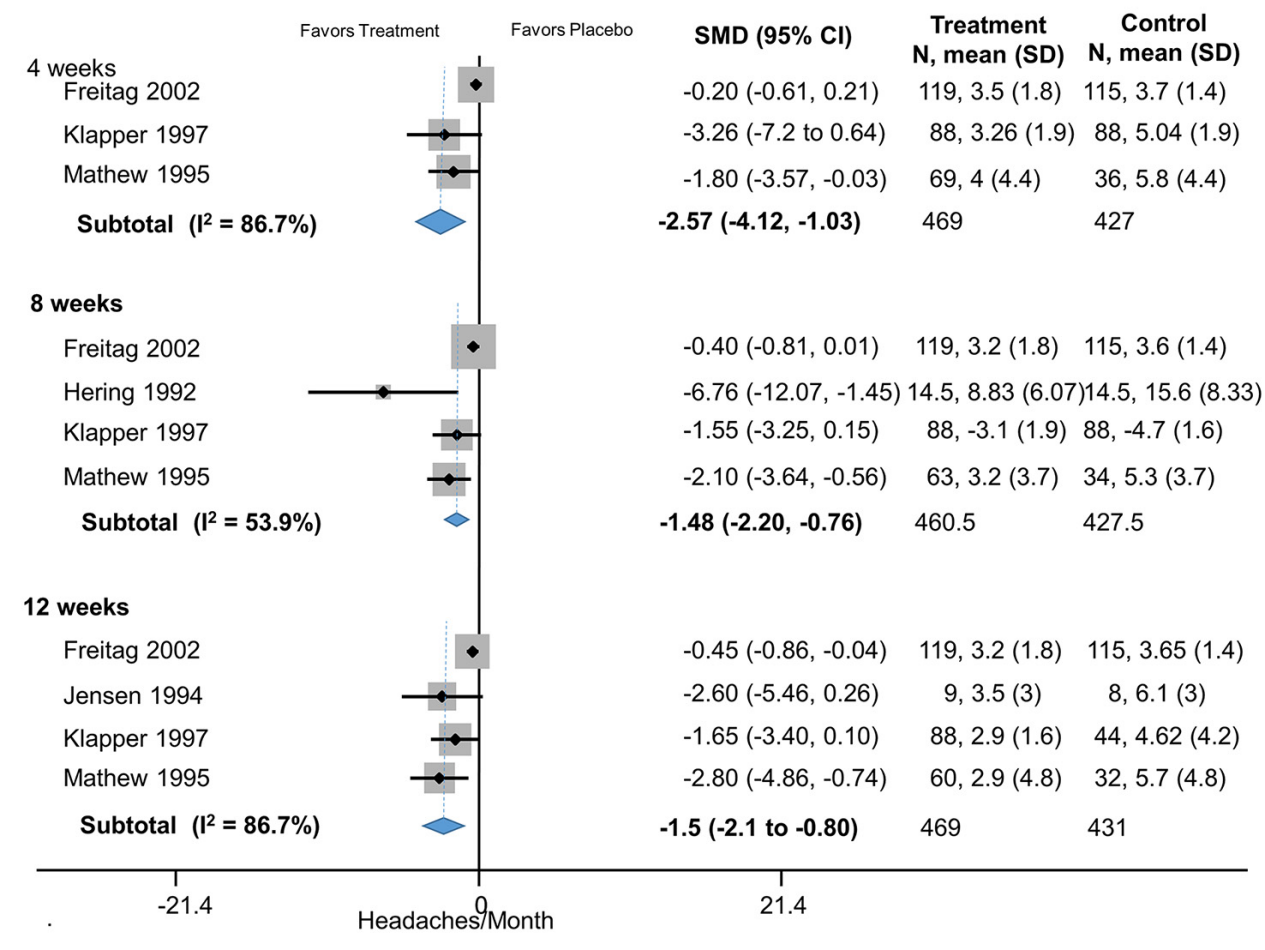
Valproate compared to placebo for episodic migraine headaches.

Anticonvulsants that were found to be more effective than placebo for episodic migraine included levetiracetam (Table 6), topiramate (Fig 4) and valproate (Fig 5). Both topiramate and valproate had numerous trials demonstrating benefit at multiple time points (Table 5).

#### Topiramate

**Fig 6:**
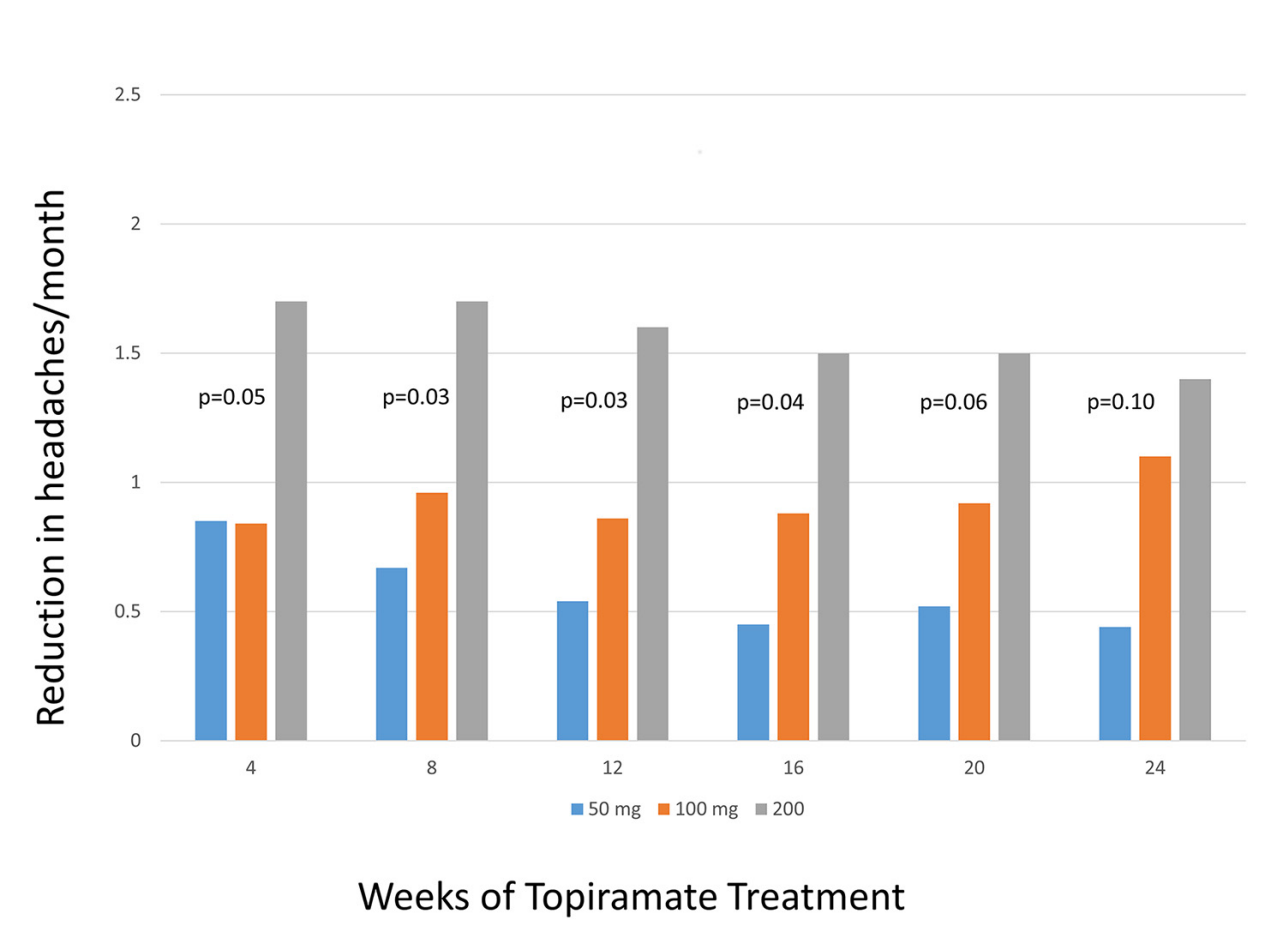
Dose response relationship of headache to topiramate dose.

Topiramate has been evaluated in twelve placebo-controlled trials that reported outcomes at numerous time points and different doses (50, 100 and 200mg). Pooled results suggest that topiramate was more effective than placebo at all time points (4–24 weeks, Table 5) and at all doses assessed. There was evidence that higher doses of topiramate was more effective than lower ones, with a stepwise increase as the dose increased from 50 to 100 to 200mg (Fig 6). For chronic migraine, 2 studies of topiramate suggested effectiveness for up to 16 weeks (Table 6). In several studies (n = 8) topiramate was also demonstrated to be more effective than placebo at reducing migraine by more than 50% (Table 7).

#### Valproate

Valproate also had been compared to placebo in six trials with multiple time points and varying doses (500-1500mg). Valproate was found to be more effective than placebo for episodic migraine at all time points assessed including 4, 8 and 12 weeks (Table 5, Fig 5). However, unlike topiramate there was no evidence of a difference in response to increased doses (dose-response p = 0.83). Valproate was also found in numerous trials (n = 5) to reduce headaches by more than 50% (Table 7).

#### Beta Blockers

There were 38 trials comparing beta-blockers to placebo with a total of 2019 participants, 37 focusing on episodic (Table 2) and 1 on chronic migraine headaches (Table 3). The average rate of withdrawals was 18%. Study duration averaged 11 weeks (range 4–64) with a mean of 64 participants (range 20–568). The majority (82%) reported headache frequency, four trials used headache index, and one duration. There were a variety of beta-blockers tested including acebutolol (n = 1), alprenolol (n = 1), atenolol (n = 3), bisoprolol (n = 1), metoprolol (n = 4), oxprenolol (n = 1), pindolol (n = 2), propranolol (n = 19) and timolol (n = 4).

**Fig 7:**
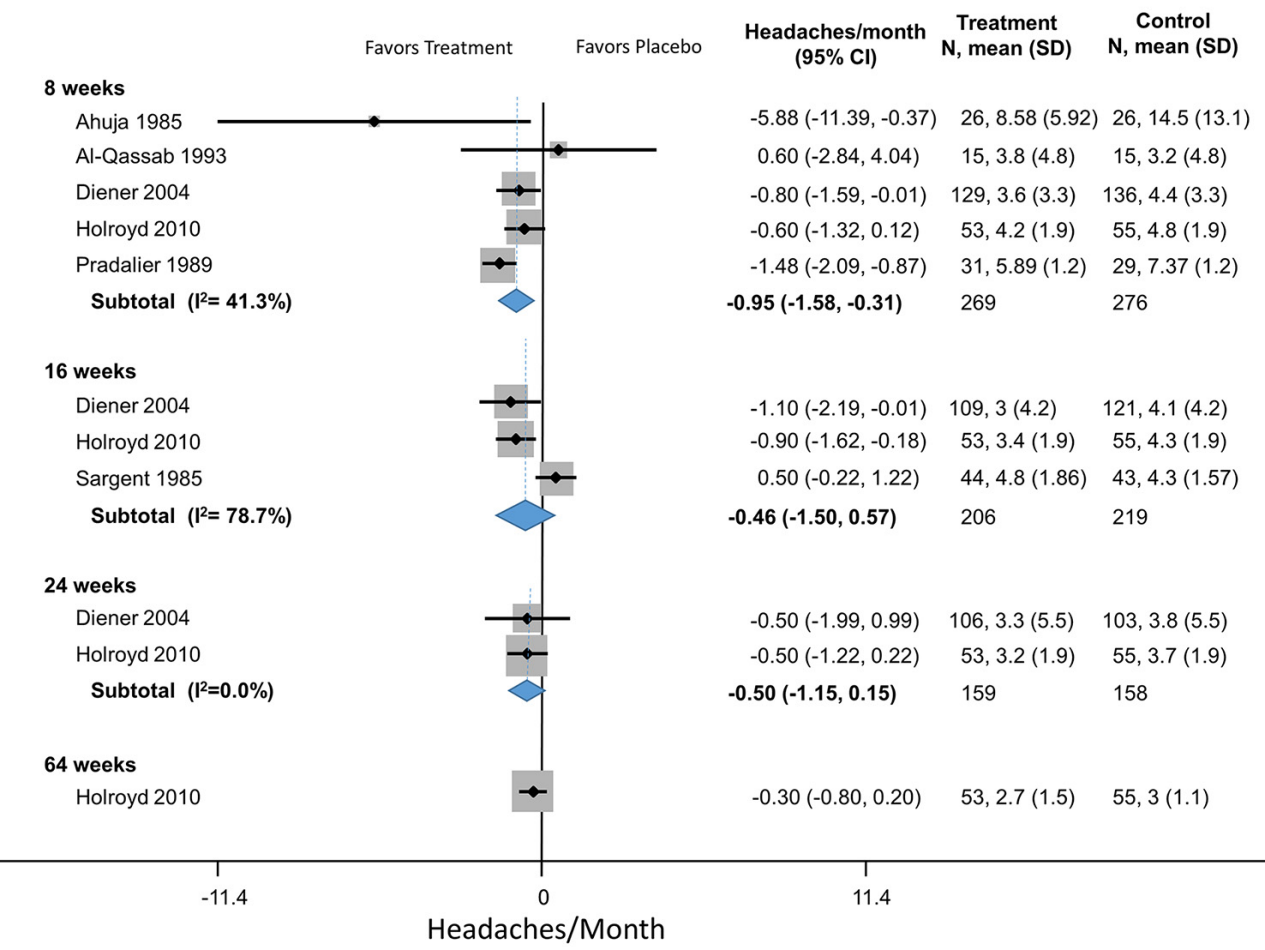
Propranolol compared to placebo for episodic migraine headaches.

Beta blockers no more effective than placebo included acebutolol, alprenolol, bisoprolol, oxprenolol and pindolol (Table 5). Beta-blockers superior to placebo for episodic migraine headaches (Table 5) included atenolol, metoprolol, propranolol (Fig 7) and timolol. Seven studies found that propranolol reduced headache by 50% (Table 7). Neither atenolol (1 study) nor propranolol (2 studies) were effective for chronic migraine (Table 6).

#### Calcium Channel Blockers

Calcium blockers headache trials tested cyclendelate (n = 1), nicardipine (n = 1), nifedipine (n = 2), nimodipine (n = 5) and verapamil (n = 2). All studies focused on episodic migraines (Table 2). Overall there were a total of 878 participants who averaged 35 years in age (range 15–65) with 78% women. The average rate of withdrawals was 18%. Study duration averaged 11 weeks (range 4–20) with a mean of 52 participants (range 12–192). No calcium channel blocker was more effective than placebo, including cyclendelate, nicardipine, nifedipine, nimodipine and verapamil (Table 5). When the dihydropyridines (nicardipine, nifedipine, nimodipine) were pooled, they were no better than placebo at reducing headaches.

#### Flunarizine

**Fig 8:**
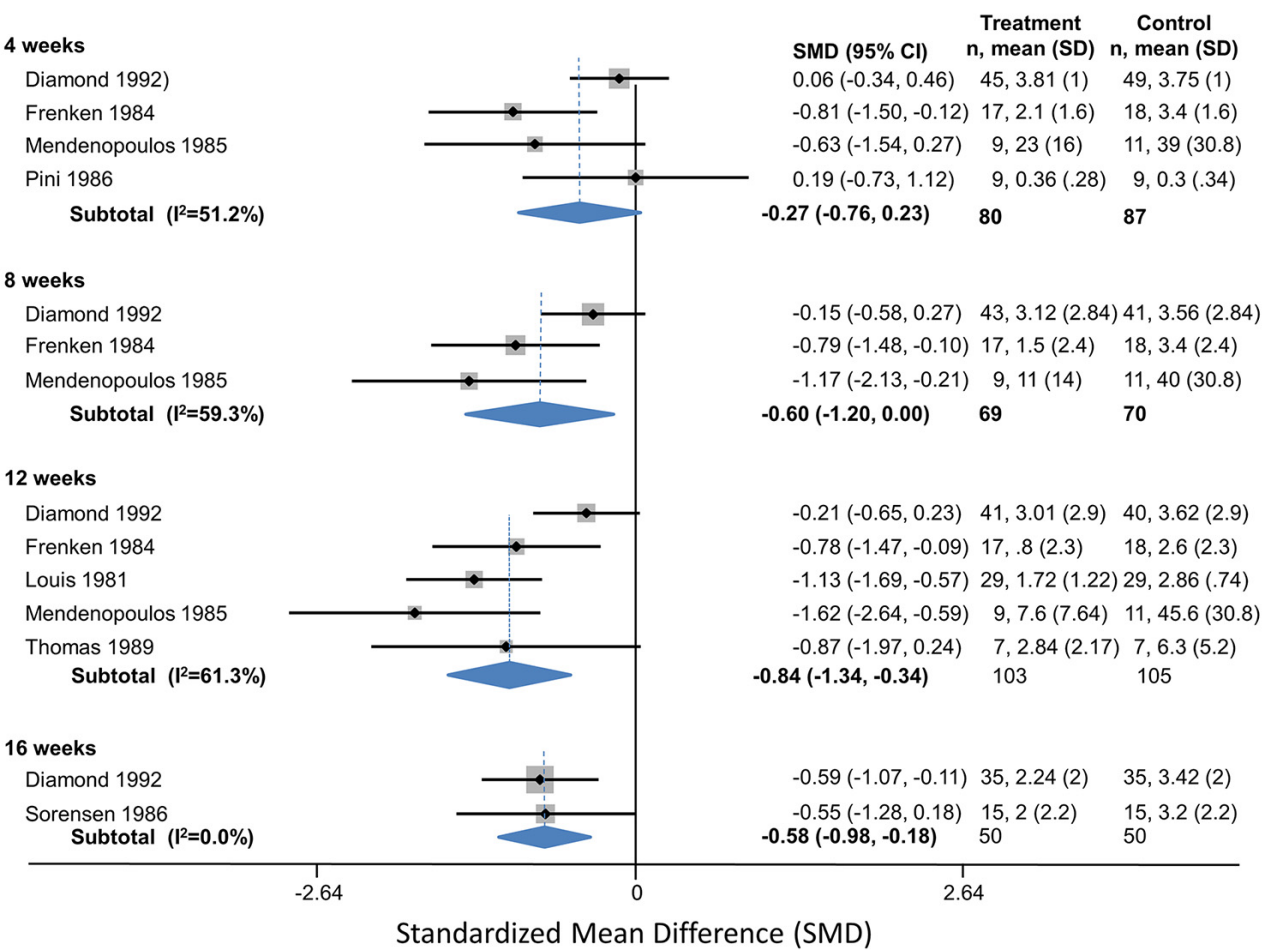
Flunarizine compared to placebo for episodic migraine headaches.

While classified as a calcium channel blocker, flunarizine has no influence on blood pressure and its side effect profile suggests that its site of action is on cellular receptors other than the calcium channel [231,232]. Flunarizine is not available in the United States. There were 7 studies of episodic migraines, totaling 332 participants (Table 2). Studies averaged 47 participants, 36.4 years in age, 77% women, 12.5 weeks in duration and 9% dropouts. Four studies reported headache frequency and three reported headache outcomes based on a headache index. Flunarizine was superior to placebo at 8 and 12 weeks (Table 5, Fig 8), though not at 4 weeks. Only a single trial reported the likelihood of a 50% reduction in headache with flunarizine with insignificant results (Table 7).

#### Selective Serotonin Reuptake Inhibitors (SSRI)/ Selective Norepinephrine Reuptake inhibitors (SNRI)

There were six SSRI and one SNRI placebo controlled trials, five focusing on migraines and 1 on chronic daily headaches. There were a total of 335 participants who averaged 36.9 years in age (range 18–65) with 81% women (Table 2). The average rate of withdrawals was 25% (range 0–41%). Study duration averaged 12 weeks (range 8–20) with a mean of 56 participants (range 27–111). Specific drugs tested include three SSRIs (femoxitine, n = 1, fluoxetine, n = 4 and sertraline, n = 1), and one SNRI (venlaxafine, n = 1). Four of the SSRI trials reported a headache index. One SSRI trial and the SNRI trial reported frequency of headaches per month.

**Fig 9:**
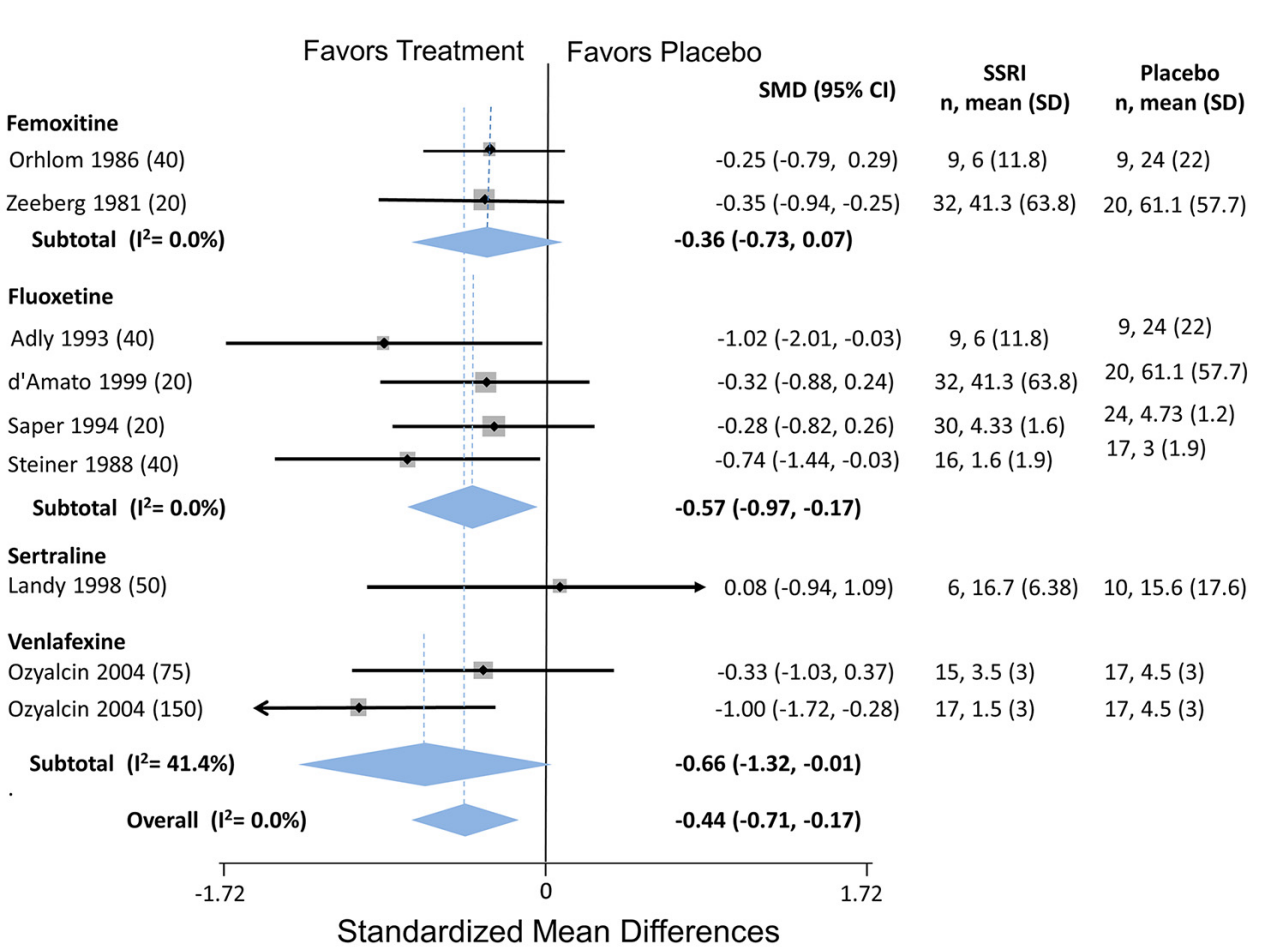
SSRI/SNRIs compared to placebo for episodic migraine headaches.

For migraine headaches, two SSRI’s, femoxitine and sertraline, were no more effective than placebo while fluoxetine was effective at 12 weeks (Fig 9). A single trial of venlafaxine found benefit at 8 weeks (Table 5). For chronic daily headache a single trial of fluoxetine found no benefit (Table 6). Only a single trial (fluoxetine) investigated the likelihood of reducing headaches by at least 50% and found no benefit over placebo (Table 7).

#### Serotonin Antagonists

**Fig 10:**
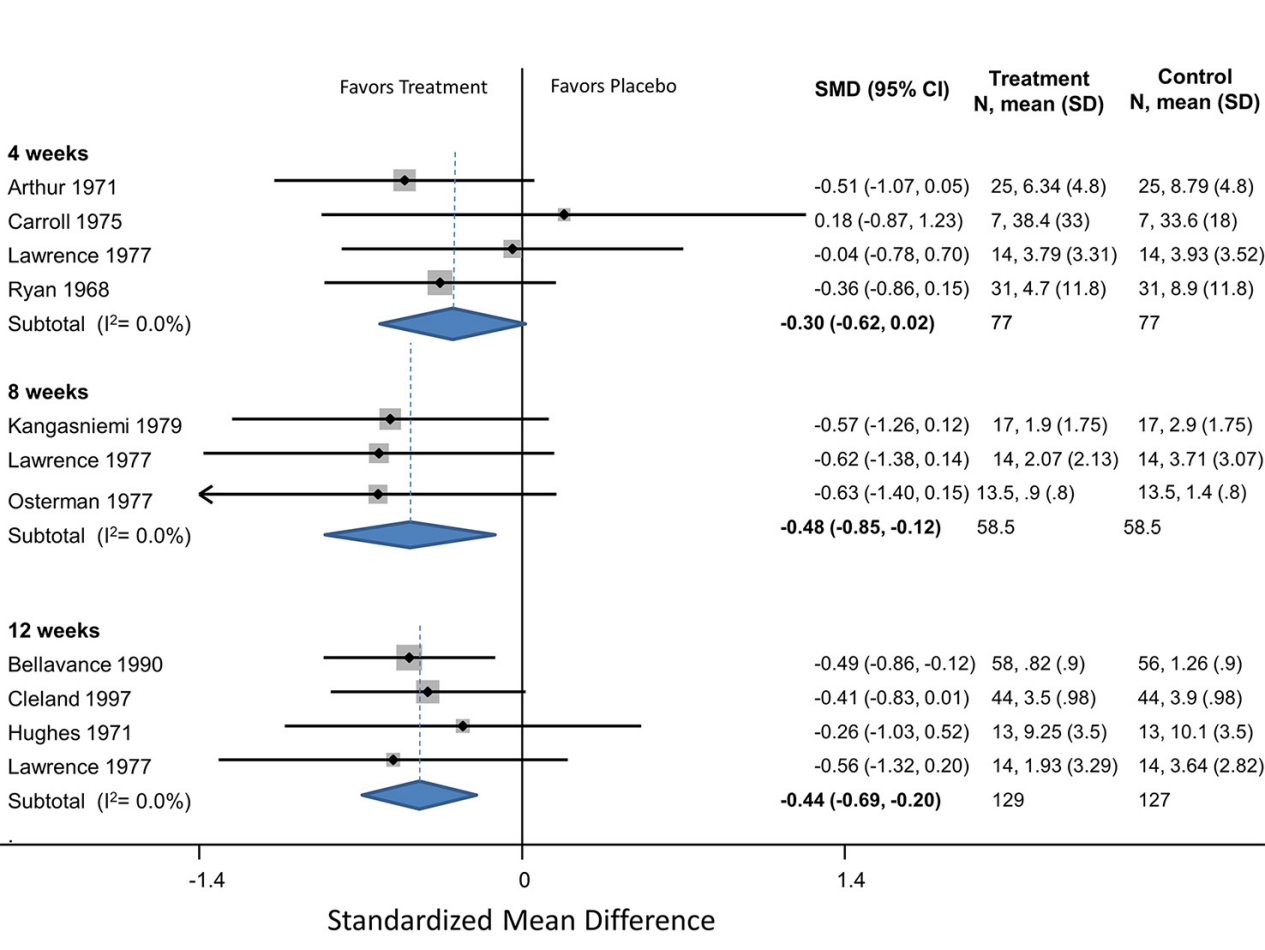
Pizotifen compared to placebo for episodic migraine headaches.

Pizotifen is a serotonin antagonist, commonly used for migraine treatment in the 1970’s and 80’s. There were 9 placebo controlled trials with a total of 600 participants and all focused on episodic migraine headaches (Table 2). The average rate of withdrawals was 20% (range 0–48). Study duration averaged 8 weeks (range 4–12) with a mean of 67 participants (range 26–176). Two studies reported a headache index, the other 7 headache frequency. Pizotifen was superior to placebo at all time points (Fig 10, Table 5). No trials reported on the likelihood of achieving at least 50% improvement in headaches.

### Tricyclic Antidepressants (TCA)

There were 8 trials comparing a TCA to placebo, one focusing on chronic daily headaches, the remainder on episodic migraine headaches. There were a total of 1570 participants. The average rate of withdrawals was 37% (range 20–52%). Study duration averaged 10 weeks (range 4–24) with a mean of 143 participants (range 10–554). Tricyclic’s studied included amitriptyline (n = 5), clomipramine (n = 2) doxepin (n = 1) and opipramol (n = 1). Four trials reported headache frequency and 4 used a headache index as their outcome measure.

**Fig 11:**
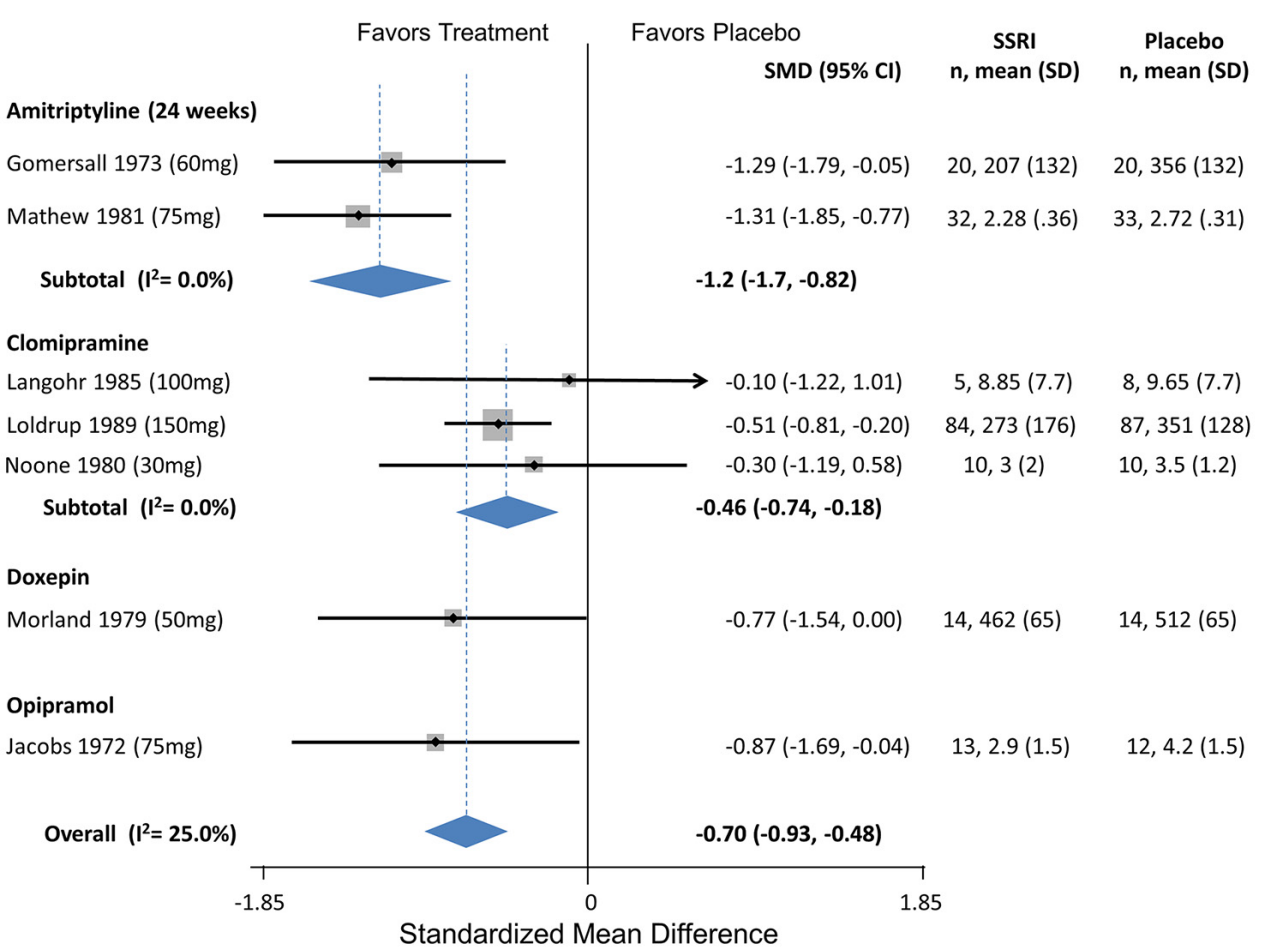
TCAs compared to placebo for episodic migraine headaches.

**Fig 12:**
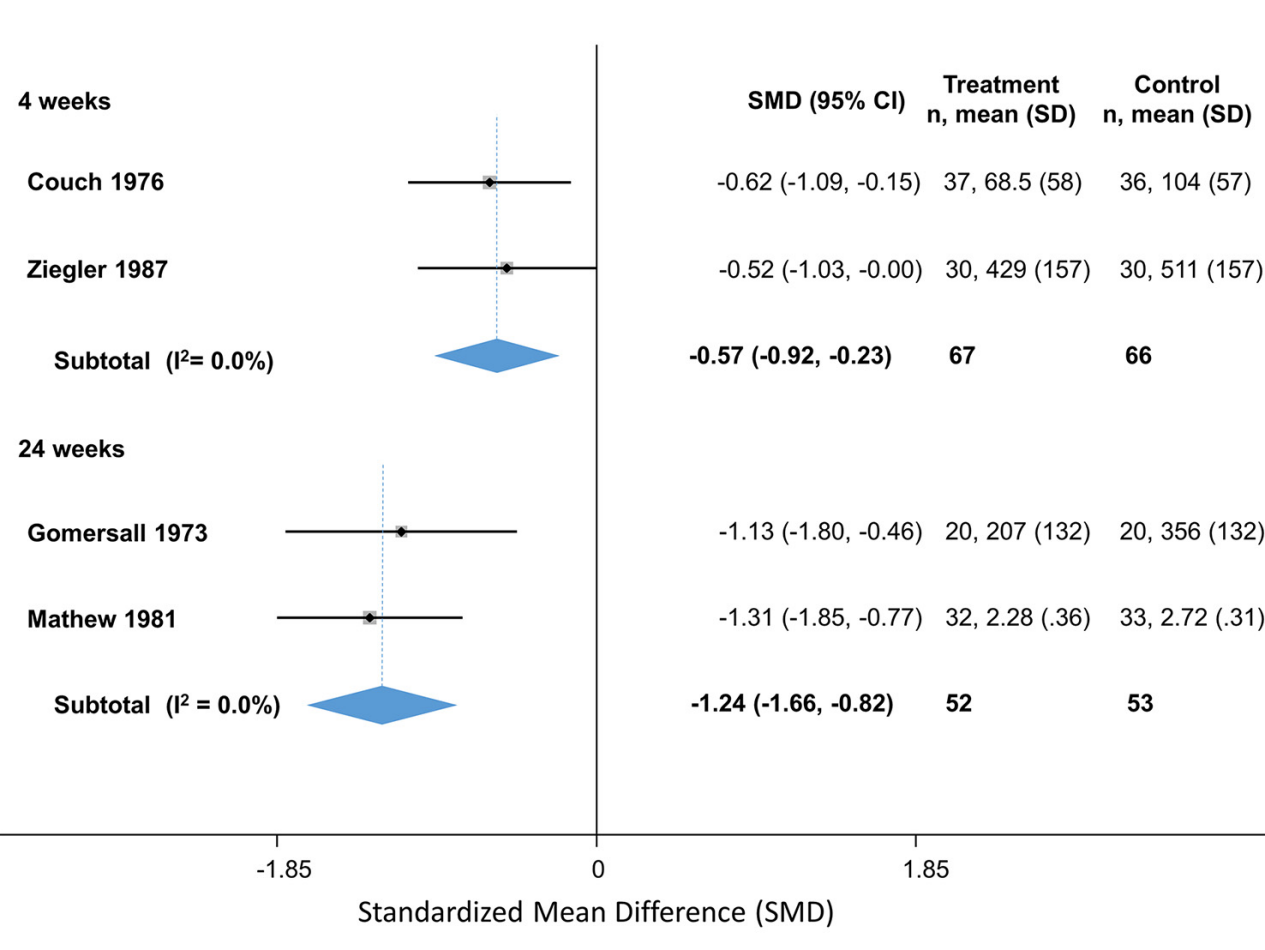
Amitriptyline compared to placebo for migraine headaches.

For episodic migraines, amitriptyline, clomipramine and doxepin were better than placebo (Table 5, Fig 11), while opipramol (Table 5) was ineffective. Amitriptyline was the best studied TCA (Fig 12), though two of the studies were only 4 weeks in duration. Amitriptyline was more likely than placebo to produce a 50% reduction in episodic migraine headaches (Table 7). A single trial found amitriptyline ineffective for chronic daily headaches (Table 6).

### Comparative Effective Trials

There were a total of 60 trials with comparisons between different prophylactic drugs for headaches, 55 including subjects with episodic headaches, five with chronic migraine headaches. Not all prophylactic drugs were directly compared with each other (Table 8). Quality ratings for these trials are given in Table 9. Drugs that were frequently compared to other active drugs include amitriptyline, metoprolol, pizotifen, propranolol, topiramate and valproate. There were few differences in effectiveness between the different drugs. Amitriptyline was no more effective than SSRIs, venlafaxine, topiramate or propranolol. Among beta-blockers, metoprolol was superior to clonidine, flunarizine and nifedipine and propranolol was better than femoxitine. Propranolol was equivalent to metoprolol, atenolol, nadolol as well as to flunarizine and topiramate (Table 10). Among the anticonvulsants, topiramate was equivalent to flunarizine, lamotrigine and to valproate and valproate was equivalent to flunarizine. For chronic migraines, popranolol was better than nortriptyline.

**Table 8:** Characteristics of comparative effectiveness trials.

Author, year, Country	Migraine Type	Baseline Headache Frequency	Comparison Drugs	Headache Measure	Study design (washout, weeks)	Duration, weeks	Sample size	Drop-outs	Age	Female
**Episodic (<15 headaches/month)**										
Afshari [180], 2012, Iran	Episodic	7.6	Topiramate (50) v. Valproate (400)	Frequency	Parallel	12	76	26%	30.7	79%
Albers [181], 1989, USA	Episodic	4.3	Propranolol (180) v. Nifedipine (90)	Frequency	Parallel	24	40	50%	35.2	85%
Amelin[182], 2000, Russia	Episodic	4.3	Amitriptyline (25) v. Fluoxetine (20)	Frequency	Parallel	12	46	23%	36	95%
Andersson [183], 1973, Denmark	Episodic	2.3	Pizotifen (2) v. Methysergide (4)	Frequency	Crossover (0)	12	73	33%	ns	84%
Andersson [242], 1981, Denmark	Episodic	5.7	Propranolol (160) vs. Femoxetine (400)	Frequency	Crossover (0)	8	49	24%	38	69%
Ashtari [185], 2008, Iran	Episodic	5.95	Propranolol (80) v. Topiramate (50)	Frequency	Parallel	8	62	3%	30.8	82%
Bank [186], 1994, Hungary	Episodic	ns	Amitriptyline (25) v. Fluvoxamine (50)	Frequency	Parallel	12	44	23%	34	80%
Bellavance [163], 1990, Canada	Episodic	6.7	Pizotifen (1.5) v. Naproxen (1100)	Frequency	Parallel	12	176	14%	32.5	79%
Bordini [190], 1997, Brazil	Episodic	3.9	Propranolol (60) v. Flunarizine (10)	Headache Index	Parallel	24	52	13%	ns	91%
Bostani [191], 2013, Iran	Episodic	6.1	Valproate (400) v. Cinnarizine (50)	Frequency	Parallel	12	132	21%	32.2	68%
Bulut [192], 2004, Turkey	Episodic	3.5	Amitriptyline (75) v Venlafaxine (150)	Frequency	Crossover (4)	12	52	32%	31.9	85%
Cady [193], 2011, USA	Episodic	5.9	Topiramate (100) v. frovatriptan (5- with aura)	Frequency	Parallel	8	55	20%	37.5	78%
Cerbo [194], 1986, Italy	Episodic	ns	Flunarizine (15) v. Pizotofen (1.5)	Frequency	Crossover (2)	8	27	33%	ns	ns
Diener [106], 1996, Germany	Episodic	4	Propranolol(120) v Cyclendalate (1200) v. Placebo	Duration (hours)	Parallel	12	214	17%	39	78%
Diener [195], 2002, EU	Episodic	3	Propranolol (160) v. Flunarizine (5) v. Flunarizine (10)	Frequency	Parallel	16	783	18%	37	81%
Diener [73], 2004, EU	Episodic	3.9	Propranolol (160) v. Topiramate (100) v. Placebo	Frequency	Parallel	26	568	37%	40.8	80%
Dodick [196], 2009, USA	Episodic	6.9	Amitriptyline (100) v. Topiramate (100)	Frequency	Parallel	26	331	43%	38.8	85%
Formisano [198], 1991, Italy	Episodic	4	Propranolol (120) v. Nimodipine (120)	Frequency	Parallel	12	22	14%	39.2	55%
Forssman [199], 1972, Sweden	Episodic	6.8	Pizotifen (3) v. methysergide (3)	Frequency	Crossover (0)	10	22	23%	40.3	53%
Gawel [200], 1992, Canada	Episodic	4.7	Propranolol (160) v. Flunarizine (10)	Frequency	Parallel	16	94	19%	35.7	90%
Gerber [201], 1991, Germany	Episodic	3.5	Propranolol (120) v. Metoprolol (200) v. Nifedipine (20)	Frequency	Parallel	12	58	28%	42.4	81%
Gupta [202], 2007, India	Episodic	6.9	Topiramate (50) v. Lamotrigine (50)	Frequency	Crossover (1)	4	57	7%	29.4	78%
Havanka-Kannianen [141], 1987, Finland	Episodic	5.2	Nimodipine (120mg) v. Pizotifen (1.5) v. Placebo	Frequency	Crossover (4)	12	43	14%	37.6	79%
Hübbe [35], 1973, Denmark	Episodic	ns	Pizotifen (1.5) v. Prochlorperazine (15) v. Placebo	Frequency	Crossover (0)	8	50	32%	35	71%
Kalita [204], 2013, India	Episodic	10.8	Amitriptyline (50) v. Valproate (1000)	Frequency	Parallel	24	300	0%	32	80
Kangasniemi [167], 1979, Finland	Episodic	6.2	Pizotifen (1.5) v. Divascan (5) v. Placebo	Frequency	Crossover (4)	7	50	13%	37	71%
Kangasniemi [205], 1983, Finland	Episodic	7.2	Propranolol (160) v. Femoxitine (400)	Frequency	Crossover (1)	16	29	17%	37	86%
Kangasniemi [116], 1984, Finland	Episodic	5.3	Propranolol (240) v. Metoprolol (200)	Frequency	Crossover (3)	8	36	8%	33.8	89%
Kaniecki [206], 1997, USA	Episodic	4.4	Dvalproex (1500) v. Propranolol (240) v. Placebo	Headache Index	Parallel	12	37	14%	ns	81%
Kass [178], 1980, Norway	Episodic	ns	Propranolol (40) v. Clonidine (0.05)	Frequency	Crossover (0)	16	23	9%	39.7	70%
Keskinbora [207], 2008, Turkey	Episodic	6.1	Amitriptyline (150) v Topiramate (200)	Frequency	Parallel	12	63	16%	37	67%
Krymchantowski [209], 2012, Brazil	Episodic	7	Topiramate (100) v. Nortriptyline (30) v. combination	Frequency	Parallel	10	38	13%	36	85%
Langohr [175], 1985, Germany	Episodic	ns	Clomipramine (100) v. Metoprolol (100) v. Placebo	Frequency	Crossover (4)	4	36	43%	44	67%
Louis [210], 1982, Belgium/Netherlands	Episodic	2.2	Flunarizine (10) v. Pizotifen (3)	Frequency	Crossover (0)	16	75	ns	37	57%
Louis [179], 1985, Belgium	Episodic	ns	Metoprolol (100) v. Clonidine (0.1)	Frequency	Crossover (2)	8	33	6%	33.5	81%
Lucking [211], 1988, Germany	Episodic	6	Propranolol (120) v Flunarizine (10)	Frequency	Parallel	16	434	23%	42	82%
Ludin [212], 1989. Switzerland	Episodic	6.3	Propranolol (120) v. Flunarizine (10)	Frequency	Parallel	16	87	17%	42	74%
Luo [213], 2012, China	Episodic	4.5	Topiramate () v. Flunarizine (5) v. combination	Frequency	Parallel	48	150	16%	43	71%
Mathew [118], 1981, USA	Unclear	ns	Amitriptyline (75) v Propranolol (160) v. Placebo	Frequency	Parallel	24	554	22%	38	95%
Mitsikostas [214], 1997, Greece	Episodic	4.6	Valproate (1000) v. Flunarizine (10)	Frequency	Parallel	8	44	ns	35.4	73%
Mohammadianinejad [215], 2011, Iran	Episodic	7.4	Topiramate (100) v. Zonisamide (200)	Frequency	Parallel	12	80	6%	34.3	69%
Olerud [216], 1986, Sweden	Episodic	4.6	Propranolol (80) v. Nadolol (80)	Frequency	Parallel	12	28	ns	ns	ns
Olsson [217], 1984, Sweden	Episodic	5.4	Propranolol (80) v. Metoprolol (100)	Frequency	Crossover (4)	8	56	5%	39	73%
Osterman [169], 1977, Sweden	Episodic		Pizotifen (0.5) v. Divascan v. Placebo	Frequency	Crossover (2)	8	30	10%	37	70%
Presthus [218], 1971, Norway	Episodic		Pizotifen (1.5) v. Methysergide (3)	Frequency	Crossover (1)	21	21	10%	42.7	67%
Rampello [219], 2004, Italy	Episodic		Amitriptyline (50) v. Citalopram (20)	Frequency	Parallel	16	88	0%	39	63%
Rascol [220], 1986, France	Episodic	4.3	Flunarizine (10) v. Pizotifen (2)	Frequency	Parallel	16	35	9%	38	71%
Ryan [170], 1968, USA	Episodic	8.5	Pizotifen (4) v. Methysergide (4) v. Placebo	Frequency	Crossover (0)	4	62	ns	ns	ns
Ryan [221], 1984, USA	Episodic	6.5	Propranolol (160) v. Nadolol (80) v. Nadolol (160)	Frequency	Parallel	12	48	6%	ns	73%
Scholz (188), 1987, Germany	Episodic		Propranolol (80) v. Metoprolol (100) v. Nifedipine (40) v. Flunarizine (10)	Frequency	Parallel	12	109	24%	40.4	83%
Shaygannejad [223], 2006, Iran	Episodic	5.4	Topiramate (50) v. Valproate (500)	Crossover (8)	Parallel	8	64	0%	34.1	60%
Shimell [224], 1990, S Africa	Episodic	4.7	Propranolol (240) v. Flunarizine (10)	Frequency	Parallel	16	57	2%	34	70%
Sorensen [225], 1991, Denmark	Episodic	4.3	Metoprolol (200) v. Flunarizine (10)	Frequency	Parallel	20	149	15%	42	79%
Stovner, 2013, Norway	Episodic		Candesartan (16), Propranolol (160)	Frequency	Crossover	12	61	15%	37	82%
Sudilovsky [226], 1987, USA	Episodic	5.3	Nadolol (80) v. Nadolol (160)	Frequency	Parallel	8	168	20%	ns	ns
Stensrud (107), 1980, Norway	Episodic	5.6	Propranolol (160) v. Atenolol (100) v. Placebo	Crossover(1)Crossover (1)	Parallel	6	7	20%	25	69%
Tarasova [227], 2008, Russia			Amitriptyline () v. Fluvoxamine ()	Frequency	Parallel					
Togha [228], 2008, Iran	Episodic	7.2	Valproate (600) v. Cinnarizine (75)	Frequency	Parallel	12	125	37%	34.1	80%
Vilming [229], 1985, Sweden/Norway	Episodic	6	Metoprolol (100) v. Pizotifen (1.5)	Frequency	Crossover (0)	4	35	ns	37.6	83%
Zain [230], 2013, Pakistan	Episodic	11.32	Topiramate (200) v. Gabapentin (1200)	Frequency	Parallel	12	80	0%	32	80%
Ziegler [136], 1987, USA	Episodic	Ns	Amitriptyline (150) v. Propranolol (240) v. Placebo	Frequency	Crossover (4)	4	30	44%	38	73%
**Chronic Migraine (≥15 headaches/month)**										
Bartolini [187], 2005, Italy	Chronic Migraine	26.6	Topiramate (75) v. Valproate (750)	Frequency	Parallel	8	49	14%	41.8	70%
Behan [188], 1986, UK	Chronic Migraine	15	Pizotifen (1.5) v. Naproxen (1100)	Frequency	Parallel	12	74	45%	ns	82%
Domingues [197], 2009, Brazil	Chronic Migraine	16.7	Nortriptyline (40) v. Propranolol (80)	Frequency	Parallel	6	76	42%	ns	ns
Krymchantowski [208], 2002, Brazil	Chronic (transformed) migraine	25.7	Amitriptyline (40) v. Amitriptyline (40)+Fluoxetine (40)	Frequency	Parallel	9	39	44%	36.4	67%
Stensrud (107), 1980, Norway	Chronic	22	Propranolol (160) v. Atenolol (100) v. Placebo	Crossover(1)Crossover (1)	Parallel	6	28	20%	25	69%

**Table 9:** Quality Assessment among comparative effectiveness trials.

			Cochrane Risk of Bias						
Study	Jadad Score (0–8)	Intention to Treat	Adequate sequence generation	Adequate concealed allocation	Adequate Blinding	Incomplete outcome data addressed	Free of selective outcome reporting	Free of “other” bias	Industry sponsored
**EPISODIC MIGRAINES**									
Afshari [180], 2012, Topiramate/valoproate	4	No	Unclear	Unclear	Unclear	No	Unclear	Unclear	No
Albers [181], 1989, Propranolol/Nifedipine	5	No	Yes	Yes	No	No	No	No	Yes
Amelin[182], 2000, Amitriptyline/Fluoxetine	4	No	No	Unclear	No	No	Unclear	Yes	No
Andersson [183], 1973, Pizotifen/Methysergide	4	No	Unclear	Unclear	Unclear	Unclear	Unclear	Unclear	Yes
Andersson [242], 1981, Propranolol/Femoxitine	4	No	Unclear	Unclear	Unclear	Unclear	Unclear	Unclear	Unclear
Ashtari [185], 2008, Propranolol/Topiramate	5	No	Yes	Yes	Unclear	No	Yes	Unclear	Unclear
Bank [186], 1994, Amitriptyiline/Fluvoxamine	4	No	Unclear	Unclear	Unclear	No	Unclear	Yes	Unclear
Bartolini [187], 2005, Topiramate/Valproate	3	No	Unclear	Unclear	Unclear	Unclear	Unclear	Unclear	Unclear
Behan [188], 1986, Pizotifen/Naproxen	2	No	Unclear	Unclear	Unclear	Unclear	Unclear	Unclear	Unclear
Bellavance [163], 1990, Pizotifen/Naproxen	3	No	Unclear	Unclear	Unclear	Unclear	Unclear	Unclear	Unclear
Bordini [190], 1997, Propranolol/Flunarizine	4	No	Unclear	Unclear	Yes	No	No	No	Unclear
Bulut [192], 2004, Amitriptyline/Venlafaxine	6	No	Unclear	Unclear	Yes	No	Unclear	Yes	Yes
Cady [193], 2011, Topiramate/Froyatriptan	3		Unclear	Unclear	No	Unclear	Unclear	Unclear	Yes
Cerbo [194], 1986, Flunarizine/Pizotifen	6	Yes	Unclear	Unclear	Yes	Unclear	Unclear	Unclear	Unclear
Diener [106], 1996, Propranolol/Cyclendalate/Placebo	4	Yes	Unclear	Unclear	Unclear	Unclear	Unclear	Unclear	Unclear
Diener [195], 2002, Propranolol/Flunarizine	8	No	Yes	Yes	Yes	No	Unclear	Unclear	Yes
Diener [73], 2004, Propranolol/Topiramate/Placebo	8	Yes	Unclear	Unclear	Unclear	Yes	Yes	Unclear	Yes
Dodick [196], 2009, Amitriptyline/Topiramate	8	No	Yes	Yes	Yes	Yes	Yes	Yes	Yes
Domingues [197], 2009, Nortriptyline/Propranolol	4	No	Unclear	Unclear	Unclear	Unclear	Unclear	Unclear	Unclear
Formisano [198], 1991, Propranolol/Nimodipine	4	No	Unclear	Unclear	No	No	No	No	Unclear
Forssman [199], 1972, Pizotifen/Methyergide	5	No	Unclear	Unclear	t	Unclear	Unclear	Unclear	Unclear
Gawel [200], 1992, Propranolol/Flunarizine	4	No	Unclear	Unclear	Unclear	No	No	Unclear	Yes
Gerber [201], 1991, Propranolol/Metoprolol/Nifedipine	3	No	Unclear	Unclear	Unclear	No	Yes	Unclear	Unclear
Gupta, 2007, Lamotrigin/Topiramate/Placebo	8	Yes	Yes	Yes	Yes	Unclear	Unclear	Unclear	Yes
Havanka-Kannianen [141], 1987, Nimodipine/Pizotifen/Placebo	3	No	Unclear	Unclear	Unclear	Unclear	Unclear	Unclear	Unclear
Hübbe [35], 1973, Pizotifen/Prochlorperazine/Placebo	3	No	Unclear	Unclear	Unclear	Unclear	Unclear	Unclear	Yes
Kangasniemi [167], 1979, Pizotifen/Divascan/Placebo	6	No	50 (32%)	37 (71%)	Unclear	Unclear	Unclear	Unclear	Unclear
Kangasniemi [205], 1983, Propranolol/Femoxitine	2	No	Unclear	Unclear	Unclear	Unclear	Unclear	Unclear	Unclear
Kangasniemi [116], 1984, Propranolol/Femoxitine/Placebo	2	No	29 (17%)	37 (86%)	Unclear	Unclear	Unclear	Unclear	Unclear
Kaniecki [206], 1997, Divalproex/Propranolol/Placebo	4	No	Unclear	Unclear	Unclear	Unclear	Unclear	Unclear	Unclear
Kaas, 1980, Norway, Propranolol/Clonidine	4	Unclear	Unclear	Unclear	Unclear	Unclear	Unclear	Unclear	No
Keskinbora [207], 2008, Amitiprytilne/Topiramate	6	No	Unclear	Yes	Yes	No	Yes	Yes	Unclear
Krymchantowski [208], 2002, Amitriptyline/Amitriptyline+Fluoxetine	5	No	Unclear	Unclear	Yes	Unclear	Unclear	Unclear	No
Krymchantowski [209], 2012, Topiramate/Nortiptyline/Combination	8	No	Yes	Yes	Yes	Unclear	Unclear	Unclear	Unclear
Langohr [175], 1985, Clomipramine/Metoprolol/Placebo	4	No	Unclear	Unclear	Unclear	No	No	Yes	Yes
Louis [210], 1982, Flunarizine/Pizotifen	4	No	Unclear	Unclear	Unclear	Unclear	Unclear	Unclear	Unclear
Louis, 1985, Metoprolol/Clonidine	4	Unclear	Unclear	Unclear	Unclear	Unclear	Unclear	Unclear	No
Lucking [211], 1988, Propranolol/Flunarizine	4	No	Unclear	Unclear	Unclear	Unclear	Unclear	Unclear	Yes
Ludin [212], 1989, Propranolol/Flunarizine	3	Yes	Unclear	Unclear	Unclear	Unclear	Unclear	Unclear	Unclear
Luo [213], 2012, Topiramate/Flunarizine/Combination	2		Unclear	Unclear	Unclear	Unclear	Unclear	Unclear	Unclear
Mathew [118], 1981, Amitriptyline/Propranolol/Placebo	2	No	Unclear	Unclear	No	No	No	Yes	Unclear
Mitsikostas [214], 1997, Valproate/Flunarizine	4	No	Unclear	Unclear	No	Unclear	Unclear	Unclear	Unclear
Mohammadianinejad [215], 2011, Topiramate/Zonisamide	6	No	Unclear	Unclear	Yes	Unclear	Unclear	Unclear	Unclear
Olerud [216], 1986, Propranolol/Nadolol	4	No	Unclear	Unclear	Yes	Unclear	Unclear	Unclear	Unclear
Olsson [217], 1984, Propranolol/Metoprolol	6	No	Unclear	Unclear	Yes	Unclear	Unclear	Unclear	Unclear
Osterman [169], 1977, Pizotifen/Divascan/Placebo	5	No	Unclear	Unclear	Yes	Unclear	Unclear	Unclear	Unclear
Presthus [218], 1971, Pizotifen/Methysergide	1	No		Unclear	Unclear	Unclear	Unclear	Unclear	Unclear
Rampello [219], 2004, Amitriptyline/Citalopram	4	Yes	Unclear	Unclear	No	Yes	Unclear	Yes	Unclear
Rascol [220], 1986, Flunarizine/Pizotifen	4	No	Unclear	Unclear	Unclear	Unclear	Unclear	Unclear	Unclear
Ryan [170], 1968, Pizotifen/Methysergide/Placebo	5	No	Unclear	Unclear	Unclear	Unclear	Unclear	Unclear	Unclear
Ryan [221], 1984, Propranolol/Nadolol	2	Yes	Unclear	Unclear	Unclear	Unclear	Unclear	Unclear	Unclear
Scholz (188), 1987, Propranolol/Metoprolol/Nifedipine/Flunarizine	3	No	Unclear	Unclear	Unclear	Unclear	Unclear	Unclear	Unclear
Shaygannejad [223], 2006, Topiramate/Valproate	4	Yes	Unclear	Unclear	Unclear	Unclear	Yes	Unclear	Unclear
Shimell [224], 1990, Propranolol/Flunarizine	6	Yes	Unclear	Unclear	Yes	No	No	Unclear	Yes
Sorensen [225], 1991, Metoprolol/Flunarizine	6	No	Unclear	Unclear	Yes	Yes	Yes	Unclear	Unclear
Stensrud (107), 1980, Propranolol/Atenolol	7	No	Unclear	Unclear	Yes	Unclear	Unclear	Unclear	Yes
Sudilovsky [226], 1987, Nadolol (two doses)	5	No	Unclear	Unclear	Yes	Unclear	Unclear	Unclear	Unclear
Togha [228], 2008, Valproate/Cinnarizine	8	Yes	Yes	Yes	Yes	Yes	Yes	Yes	Unclear
Vilming [229], 1985, Metoprolol/Pizotifen	4	No	Unclear	Unclear	Unclear	Unclear	Unclear	Unclear	Unclear
Ziegler [136], 1987, Amitriptyline/Propranolol/Placebo	3	No	Unclear	Unclear	Unclear	No	Unclear	Yes	No

**Table 10:** Comparative Effectiveness Trial Outcomes.

Drug 1	Drug 2	Study (year)	Standardized Mean Difference (95% CI)δ	Heterogeneity
**Episodic Migraines (<14 headaches/month)**				
Amitriptyline	Fluoxetine	Amelin (2000)	-0.14 (-0.85 to 0.58)	
Amitriptyline	Fluvoxamine	Bank (1994)	0.37 (-0.20 to 0.93)	
**Amitriptyline**	**SSRI**	**Pooled SMD**	**0.17 (-0.32 to 0.65)**	Q = 1.15, df = 1. I^2^ = 12.9%
Amitriptyline	Maprotiline	Amelin (2000)	-0.15 (-0.87 to 0.57)	
Amitriptyline	Topiramate	Dodick (2009)	-0.08 (-0.30 to 0.13)	
Amitriptyline	Topiramate	Keskinbora (2008)	0.31 (-0.30 to 0.92)	
**Amitriptyline**	**Topiramate**	**Pooled SMD**	**0.01 (-0.32 to 0.33)**	Q = 1.41, df = 1, I^2^ = 29.2%
Amitriptyline	Venlafaxine	Bulut (2004)	-0.12 (-0.51 to 0.26)	
Amitriptyline	Propranolol	Ziegler (1987)	0.17 (-0.55 to 0.88)	
Flunarizine	Flunarizine + Topiramate	Luo (2012)	0.21 (-0.23 to 0.64)	
Metoprolol	Flunarizine	Scholz (1981)	-0.83 (-1.65 to -0.01)	
Metoprolol	Flunarizine	Sorensen (1991)	-0.35 (-0.69 to -0.02)	
**Metoprolol**	**Flunarizine**	**Pooled SMD**	**-0.43 (-0.77 to -0.10)**	Q = 1.06, df = 1. I^2^ = 5.8%
Metoprolol	Nifedipine	Gerber (1991)	-0.66 (-1.31 to -0.01)	
Metoprolol	Nifedipine	Scholz (1987)	-0.92 (-1.78 to -0.06)	
**Metoprolol**	**Nifedipine**	**Pooled SMD**	**-0.75 (-1.27 to -0.24)**	Q = 0.24, df = 1, I^2^ = 0.0%).
Metoprolol	Clomipramine	Langohr (1985)	-1.4 (-2.8 to 0.03)	
Metoprolol	Clonidine	Louis (1985)	-0.54 (-1.07 to -0.01)	
Metoprolol	Pizotifen	Vliming (1985)	-0.43 (-1.15 to 0.30)	
Pizotifen	Flunarizine	Cerbo (1986)	0.19 (-0.74 to 1.12)	
Pizotifen	Flunarizine	Louis (1982)	0.14 (-0.34 to 0.63)	
Pizotifen	Flunarizine	Rascol (1986)	0.40 (-0.29 to 1.08)	
**Pizotifen**	**Flunarizine**	**Pooled SMD**	**0.22 (-0.14 to 0.59)**	Q = 0.36, df = 2, I^2^ = 0.0%
Pizotifen	Divascan	Osterman (1977)	-0.38 (-1.14 to 0.39)	—
Pizotifen	Methysergide	Andersson (1973)	-0.17 (-0.74 to 0.40)	
Pizotifen	Methysergide	Forsmann (1972)	0.10 (-0.66 to 0.85)	
Pizotifen	Methysergide	Presthus (1971)	0.24 (-0.64 to 1.12)	
Pizotifen	Methysergide	Ryan (1968)	-0.13 (-0.63 to 0.37)	
**Pizotifen**	**Methysergide**	**Pooled SMD**	**-0.06 (-0.37 to 0.26)**	Q = 0.83, df = 3, I^2^ = 0.0%
Pizotifen	Nimodipine	Havanka (1987)	0.11 (-0.59 to 0.71)	—
Pizotifen	Naproxen	Bellavance (1990)	0.10 (-0.27 to 0.46)	—
Pizotifen	Prochloperazine	Hübbe (1973)	-0.33 (-0.96 to 0.29)	—
Propranolol	Atenolol	Stensrud (1980)	0.02 (-0.84 to 0.88)	—
Propranolol	Clonidine	Kaas (1980)	0.03 (-0.58 to 0.63)	—
Propranolol	Cyclandelate	Diemer (1996)	-0.07 (-0.38 to 0.24)	—
Propranolol	Femoxetine	Andersson (1981)	-0.40 (-1.05 to 0.25)	
Propranolol	Femoxetine	Kangasniemi (1983)	-2.03 (-2.66 to -1.39)	
**Propranolol**	**Femoxetine**	**Pooled SMD**	**-1.21 (-2.8 to -0.37)**	Q = 17.35, df = 1, I^2^ = 88.5%
Propranolol	Flunarizine	Bordini (1997)	-0.32 (-0.40 to 1.05)	
Propranolol	Flunarizine	Diener (2002)	-0.38 (-0.52 to -0.24)	
Propranolol	Flunarizine	Gawel (1992)	0.58 (0.12 to 1.04)	
Propranolol	Flunarizine	Lucking (1988)	-0.20 (-0.67 to 0.27)	
Propranolol	Flunarizine	Ludin (1989)	-0.21 (-0.73 to 0.30)	
Propranolol	Flunarizine	Scholz (1987)	-0.37 (-1.16 to 0.43)	
Propranolol	Flunarizine	Shimell (1990)	-0.02 (-0.55 to 0.50)	
**Propranolol**	**Flunarizine**	**Pooled SMD**	**-0.04 (-0.34 to 0.26)**	Q = 20.62, df = 6, I^2^ = 70.9%
Propranolol	Metoprolol	Olsson (1984)	0.00 (-0.46 to 0.46)	
Propranolol	Metoprolol	Scholz (1987)	0.03 (-0.58 to 0.65)	
**Propranolol**	**Metoprolol**	**Pooled SMD**	**0.15 (-0.27 to 0.57)**	Q = 1.14, df = 1, I^2^ = 12.6
Propranolol	Nadolol	Olerud (1986)	0.37 (-0.39 to 1.13)	
Propranolol	Nadolol	Ryan (1984)	-0.42 (-1.15 to 0.27)	
Propranolol	Nadolol	Sudilovsky (1987)	0.28 (-0.08 to 0.64)	
**Propranolol**	**Nadolol**	**Pooled SMD**	**0.19 (-0.18 to 0.56)**	Q = 2.81, df = 2, I^2^ = 28.9%
Propranolol	Nifedipine	Albers (1989)	0.84 (-0.12 to 1.79)	
Propranolol	Nifedipine	Gerber (1991)	-0.63 (-1.30 to 0.05)	
Propranolol	Nifedipine	Scholz (1987)	-0.46 (-1.29 to 0.37)	
**Propranolol**	**Nifedipine**	**Pooled SMD**	**-0.14 (-0.98 to 0.71)**	Q = 10.41, df = 2, I^2^ = 61.6%
Propranolol	Nimodipine	Formisano (1991)	-0.19 (-1.10 to 0.73)	—
Propranolol	Topiramate	Ashtari (2008)	-0.24 (-0.27 to 0.75)	
Propranolol	Topiramate	Diener (2004)	0.12 (-0.08 to 0.32)	
Propranolol	Topiramate	**Pooled SMD**	**-0.02 (-0.30 to 0.33)**	Q = 1.65, df = 2, I^2^ = 39.5%
Topiramate	Flunarizine	Luo (2012)	0.23 (-0.07 to 0.53)	
Topiramate	Frovatriptan (abortive)	Cady (2011)	-0.49 (-1.09 to 0.11)	
Topiramate	Topiramate+Flunarizine	Luo (2012)	0.35 (-0.07 to 0.78)	
Topiramate	Lamotrigine	Gupta (2007)	-0.30 (-0.83 to 0.22)	
Topiramate	Topiramate + Nortriptyline	Krymchantowski (2012)	0.53 (0.04 to 1.02)	
		Afshari (2012)	-0.32 (-0.85 to 0.20)	
		Shaygannejad (2006)	-0.19 (-0.68 to 0.30)	
Topiramate	Valproate	**Pooled SMD**	**-0.28 (-0.70 to 0.15)**	Q = 0.09, df = 1, I^2^ = 0.0%
Topiramate	Zonisamide	Mohammadianinejad (2011)	-0.26 (-0.72 to 0.19)	
Valproate	Cinnarizine	Togha (2008)	-0.07 (-0.42 to 0.28)	
Valproate	Flunarizine	Mitsikostas (1997)	-0.06 (-0.67 to 0.56)	
**Chronic Migraine (>15 headaches/month)**				
Amitriptyline	Amitriptyline+Fluoxetine	Krymchantowski (2002)	-0.44 (-1.20 to 0.33)	
Pizotifen	Naproxen	Behan (1986)	0.08 (-0.56 to 0.73)	
Propranolol	Atenolol	Stensrud (1980)	0.08 (-1.40 to 1.56)	
Propranolol	Nortriptyline	Domingues (2009)	-0.83 (-0.06 to -1.61)	
Topiramate	Valproate	Bartolini (2005)	-0.13 (-0.72 to 0.46)	

δ negative number favors drug 1, positive number favors drug 2 in these comparisons.

### Network Meta-analysis

**Fig 13:**
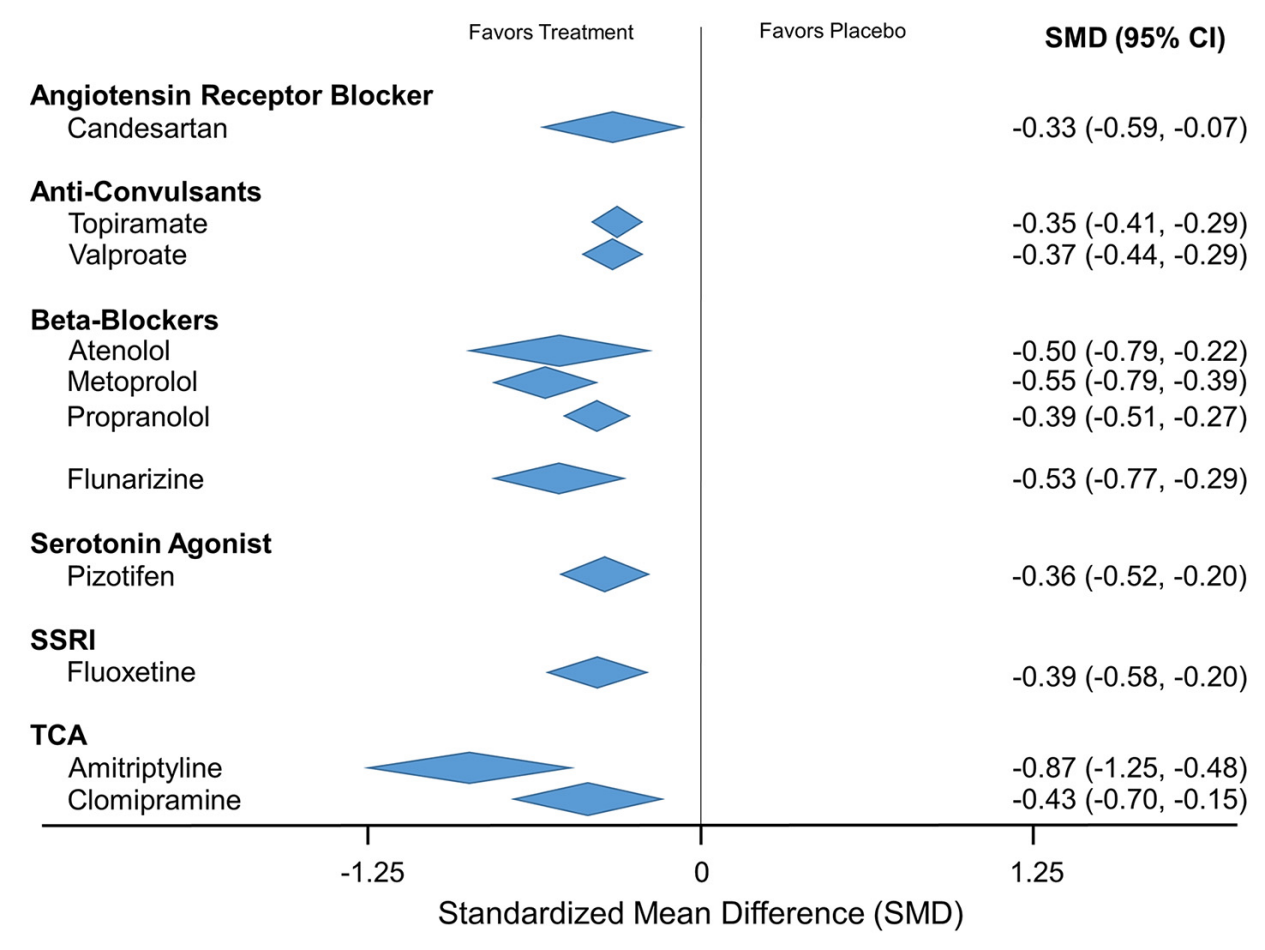
Network meta-analysis

Candidate drugs for the network meta-analysis were those drugs found effective for treatment of episodic migraine headaches with at least 3 randomized clinical trials. These included eleven different drugs used in prophylaxis of episodic migraine headaches (Fig 13). Indirect comparisons of these eleven individual drugs using meta-regression suggested that amitriptyline was more effective than several of the other drugs including candesartan (p = 0.04), fluoxetine (p = 0.03), propranolol (p = 0.009), topiramate (p = 0.005) and valproate (p = 0.009, Fig 12), and no different than atenolol (p = 0.20), flunarizine (p = 0.06), clomipramine (p = 0.15) or metoprolol (p = 0.15). The network meta-analysis found no differences between the other drugs in the relative effectiveness in the prophylaxis against migraine headaches. (p = 0.21).

### Placebo effect

**Fig 14:**
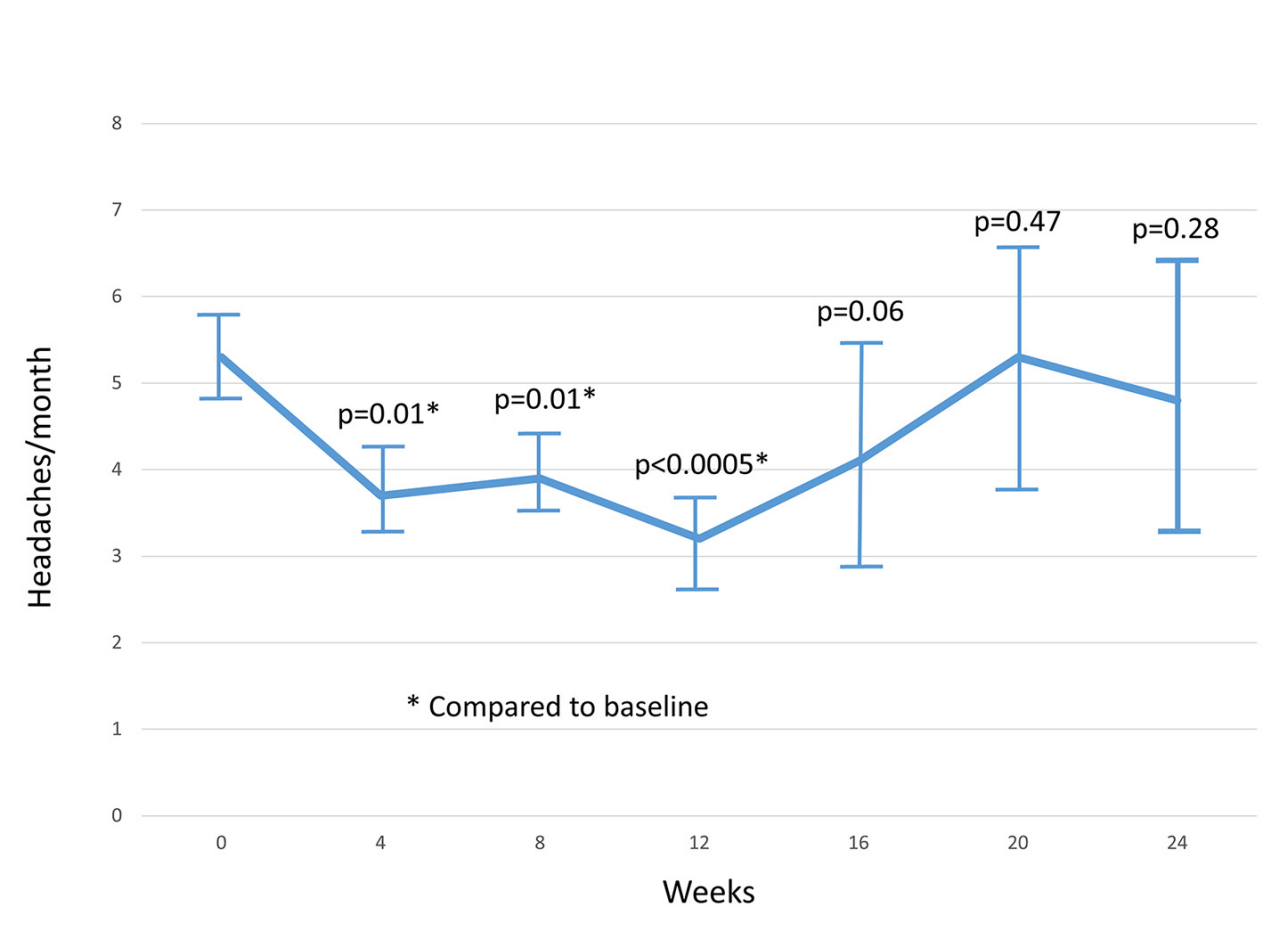
Placebo effect of treatment of episodic migraine headaches.

There were 78 studies that provided baseline headache frequency that included 4579 episodic migraine sufferers who were randomized to placebo. On average, patients randomized to the placebo group experienced 5.3 (95% CI: 4.9–5.8) headaches/month at baseline. Patients receiving placebos experienced a significant decline in headache frequency by 4 weeks, an effect that persisted through 12 weeks. By weeks 16, 20 and 24, the number of headaches experienced by patients given placebo increased back to values that were not different than baseline (Fig 14).

### Side Effects

Patients receiving prophylactic treatment were more likely than those receiving placebo to experience side effects (RR: 1.27, 95% CI: 1.19 to 1.37) and to withdraw from treatment (RR: 1.18, 95% CI: 1.08–1.29). The specific side effects varied by study medication (Table 11). Drowsiness was the most common side effect, increased among patients taking gabapentin, pizotifen, topiramate, TCA and valproate. Tricyclic antidepressants also caused dry mouth and weight gain. Beta-blockers were associated with feeling depressed, dizzy and insomnia. Topiramate increased rates of nausea and paresthesia. Pizotifen had marked increased rates of weight gain with participants averaging 4.3 kg (95% CI: 3.0–5.6).

**Table 11:** Side Effects Compared with Placebo.

	Alpha Blockers	Anti-convulsants	Beta Blockers	Calcium Channel Blockers	Flunarizine	SSRI	TCA
**“Any” side effect**	1.25 (0.91–1.71)	1.20 (1.14–1.27)	1.65 (1.41–1.93)	1.25 (1.03–1.53)	1.26 (0.67–2.37)	1.0 (0.51–1.97)	1.54 (1.37–1.74)
**Withdrawal**	1.07 (0.65–1.78)	1.23 (1.13–1.34)	1.29 (1.03–1.61)	1.14 (0.84–1.57)	1.0 (0.3–3.2)	1.13 (0.79–1.61)	1.53 (1.27–1.85)
**Depression**	3.0 (0.13–70.6)	ns	4.1 (1.1–15.2)	0.2 (0.01–4.0)	0.7 (0.1–3.4)	ns	ns
**Dizziness**	1.80 (0.36–9.08)	1.61 (1.16–2.21)	1.75 (1.04–2.95)	1.19 (0.45–3.18)	ns	1.28 (0.23–7.14)	1.20 (0.77–1.86)
**Fatigue**	2.65 (0.94–7.51)	2.22 (1.67–2.96)	1.19 (0.90–1.56)	3.07 (1.26–7.48)	1.3 (0.7–2.1)	ns	1.84 (1.25–2.71)
**Dry Mouth**	7.09 (2.31–21.7)	2.33 (0.43–12.8)	ns	0.21 (0.01–4.27)	0.26 (0.03–2.3)	ns	2.32 (1.63–3.28)
**Nausea/vomiting**	1.50 (0.27–8.3)	1.44 (1.01–2.03)	1.8 (1.05–3.02)	0.68 (0.37–1.24)	0.12 (0.01–2.0) (1 study)	2.15 (0.80–5.8)	1.18 (0.42–3.3)
**Parasthesias**	6.2 (1.5–26.3)	4.2 (2.7–6.6)	1.4 (0.49–4.2)	5.0 (0.25–101.9)	ns	ns	1.5 (0.26–9.0) (1 study)
**Sleep disturbance**	Ns	0.84 (0.53–1.33)	1.64 (1.08–2.5)	Ns	ns	1.27 (0.66–2.5)	0.63 (0.36–1.1)
**Weight gain**	Ns	1.02 (0.12–8.5)	6.1 (0.73–51.3)	3.08 (0.60–15.9)	0.79 (0.36–1.71) (4 studies)	ns	1.65 (1.02–3.04)

Network meta-analysis and direct comparisons found no difference in likelihood of experiencing “any” side effect or in the rate of withdrawing from studies.

### Sensitivity Analysis

There was evidence of publication bias for beta-blockers (Egger p = 0.02), and for each of topiramate (p = 0.001) and valproate (p = 0.04). There was no evidence of publication bias for the remaining drugs or classes. The metatrim test reduced the effect estimate four these four drugs, though only for valproate did the adjusted effect become insignificant (beta-blocker SMD: -0.24, 95% CI: -0.45 to -0.04; topiramate: SMD: -0.35, 95% CI: -0.57 to -0.12; valproate: SMD: -0.40, 95% CI: -0.90 to 0.10).

There were a number of quality problems (Tables 4 and 9). However, total Jadad score (p = 0.51), intention to treat (p = 0.84), sequence generation (p = 0.47), concealed allocation (p = 0.18), blinding (p = 0.84) or industry sponsorship (p = 0.17) had no relationship or impact on pooled outcomes.

The amount of heterogeneity varied considerably among the various drugs and drug classes. Longer duration of treatment was associated with greater effects for tricyclic antidepressants (β = -0.06, 95% CI: -0.09 to -0.03) as well as for valproate (β = -0.02, 95% CI -0.04 to -0.01) and flunarizine (β = -0.03, 95% CI -0.07 to -0.001). The other treatment options did not appear to be time-sensitive. There was no relationship between type of measurement (frequency vs. headache index) and outcomes (p = 0.72). Age, percent women, sample size, dropout rate, percent of maximum dose attained, study design and whether or not depressed patients were allowed to participate had no relationship with outcomes.

## Discussion

There has long been consensus that some drugs are useful in prophylaxis against migraine headaches. Our review confirms that there is good evidence for amitriptyline, atenolol, flunarizine, fluoxetine, metoprolol, pizotifen, propranolol, timolol, topiramate and valproate in reducing episodic migraine headache. At baseline, episodic migtaine sufferers averaged slightly over six headaches per month and most drugs reduced the number of headaches by 1 or 2 per month. Amitriptyline had the greatest benefit and while the network meta-analysis suggested that it was the most effective drug for preventing migraine headaches, this was not confirmed in clinical trials in which amitriptyline was directly compared with other drugs (including SSRIs, topiramate and propranolol), though all candidate drugs have not been included. Beta-blockers (atenolol, propranolol, timolol), anticonvulsants (topiramate, valproate), flunarizine and pizotifen had moderate benefit in reducing headache burden while the serotonin reuptake inhibitors had a small effect.

On average, across the effective prophylactic medications, migraine sufferers had about twice the chance of experiencing at least a 50% reduction in headaches as those receiving placebo. Our pooled risk reduction (ARR: 0.15, 95% CI: 0.09–0.21) suggests that 7 people would need to be treated to produce 50% reduction in headache burden in one subject. Side effects were common, but were predictable based on the drug mechanisms of action and are well-known.

There was a significant placebo effect that was seen within 4 weeks of placebo initiation with a gradual increase in the benefit of placebo on headaches through 12 weeks. By week 16, patients randomized to placebo had a gradual increase in the number of headaches experienced with no difference from baseline through 24 weeks of treatment. This is similar to the placebo effect we saw in our meta-analysis of pediatric migraine trials [233]. Uncontrolled trials of drugs for treatment of migraine headaches are still published, our data reinforces the importance of placebo controls.

Our study is the first to pool all the data from the numerous randomized controlled clinical trials to explore potential differences for both continuous and dichotomous outcomes and for both episodic and chronic migraine headaches. We also avoid a common error found in previous meta-analyses in which researchers pooled the outcome at the end of the study, regardless of the time point. This inappropriately pooled studies of different treatment durations.

There have been no previous systematic reviews of ACE/ARB, flunarizine or beta-blockers other than propranolol for migraine headaches. A recent Neurology Academy review was limited by several factors: 1) it included only studies since 2009, 2) it provided only qualitative statements about the level of evidence with no formal pooling of data and 3) it had no comparative effectiveness data [27]. While our findings are similar to previous reviews of anticonvulsants [234], the beta-blocker propranolol [235], anticonvulsants [236] and tricyclic antidepressants [237], we found some important differences. Anticonvulsants were less effective than a 2004 Cochrane review[234], though our review includes nearly twice as many studies. A 2004 Cochrane beta-blocker review included exclusively propranolol, while we include all beta-blockers. Our 2010 TCA review[237] inappropriately pooled both migraine and tension headaches together. Our 1996 review [238] also combined migraine and tension headaches, likely inappropriate given potentially important pathophysiologic differences. A 2005 Cochrane review of SSRIs found no benefit[239], but that trial was largely based on tension headaches and it also combined both migraine and tension headaches in their pooled analysis. In contrast, our larger review focuses on migraine headaches and suggests a modest effect from fluoxetine. To date, there have been no quantitative systematic reviews comparing the different classes of treatment, though one recent qualitative systematic review concluded that the choice should be tailored to patients based on side effects and comorbidities [240].

A recent systematic review examined the efficacy of prophylactic treatment for episodic migraine headaches[28] in reducing headaches by 50%, a dichotomous outcome. Our study includes both continuous and dichotomous outcomes and examines the effects for both episodic and migraine headaches. That study was limited to English language only and includes a smaller number of studies than this analysis. Our results are similar and in agreement with their conclusion that there is no difference in efficacy between the different drugs; however we found that the benefit for most drugs was less than they reported.

Our study has a number of important limitations. First the pooled differences between the various drugs and classes suggested important clinical differences. Some drugs had a large effect in headache reduction, others only small or modest ones. Our network meta-analysis suggested superiority for amitriptyline, a finding not confirmed in head-head trials. While there have been 51 trials directly comparing different drugs, these comparisons have been somewhat haphazard and many important potential comparisons have not been made.

## Conclusions

Our data suggests that the current practice of tailoring prophylactic medication according to patient characteristics and expected side effects is a good approach. Patients with migraine headaches and hypertension should consider trials with a beta blocker. Patients with depression may benefit from either SSRI or TCA. Patients with restless leg syndrome or another indication for an anticonvulsant may benefit from topiramate or valproate. Our analysis suggests that amitryptyline is more effective than the other medications, this has not been confirmed in the limited number of direct comparative effectiveness trials that have been conducted. The placebo effect, that lasts through at least 12 weeks in our study, suggests that non-placebo controlled trials should not be performed. Nearly all studies of headache treatment were 24 weeks or less in duration, this is an important limitation since migraine is a chronic condition. Whether treatment benefit persists, increases or wanes is unknown and deserving of further studies. The paucity of head-to-head comparative effectiveness trials between some classes of medication also indicates a direction for future headache research.

## Supporting Information

S1 File PRISMA Checklist. (DOC)
